# Crystal Structure of Inhibitor-Bound GII.4 Sydney 2012 Norovirus 3C-Like Protease

**DOI:** 10.3390/v15112202

**Published:** 2023-10-31

**Authors:** Alice-Roza Eruera, Alice M. McSweeney, Geena M. McKenzie-Goldsmith, Helen K. Opel-Reading, Simone X. Thomas, Ashley C. Campbell, Louise Stubbing, Andrew Siow, Jonathan G. Hubert, Margaret A. Brimble, Vernon K. Ward, Kurt L. Krause

**Affiliations:** 1 Department of Microbiology and Immunology, School of Biomedical Sciences, University of Otago, P.O. Box 56, Dunedin 9054, New Zealand; alice.eruera@otago.ac.nz (A.-R.E.); alice.mcsweeney@otago.ac.nz (A.M.M.); geena.mckenzie-goldsmith@postgrad.otago.ac.nz (G.M.M.-G.); simoneximeng@gmail.com (S.X.T.); 2 Department of Biochemistry, School of Biomedical Sciences, University of Otago, P.O. Box 56, Dunedin 9054, New Zealand; helen.opel-reading@otago.ac.nz (H.K.O.-R.); ashley.campbell@otago.ac.nz (A.C.C.); 3 School of Chemical Sciences, The University of Auckland, 23 Symonds Street and 3b Symonds Street, Auckland 1142, New Zealand; louise.stubbing@gmail.com (L.S.); asio616@aucklanduni.ac.nz (A.S.); jonathanghubert@gmail.com (J.G.H.); m.brimble@auckland.ac.nz (M.A.B.)

**Keywords:** norovirus, 3C-like protease, X-ray structure, antiviral, protease inhibitor, ligand-free protease

## Abstract

Norovirus is the leading cause of viral gastroenteritis worldwide, and there are no approved vaccines or therapeutic treatments for chronic or severe norovirus infections. The structural characterisation of the norovirus protease and drug development has predominantly focused upon GI.1 noroviruses, despite most global outbreaks being caused by GII.4 noroviruses. Here, we determined the crystal structures of the GII.4 Sydney 2012 ligand-free norovirus protease at 2.79 Å and at 1.83 Å with a covalently bound high-affinity (IC_50_ = 0.37 µM) protease inhibitor (NV-004). We show that the active sites of the ligand-free protease structure are present in both open and closed conformations, as determined by their Arg112 side chain orientation. A comparative analysis of the ligand-free and ligand-bound protease structures reveals significant structural differences in the active site cleft and substrate-binding pockets when an inhibitor is covalently bound. We also report a second molecule of NV-004 non-covalently bound within the S4 substrate binding pocket via hydrophobic contacts and a water-mediated hydrogen bond. These new insights can guide structure-aided drug design against the GII.4 genogroup of noroviruses.

## 1. Introduction

Human norovirus (HuNoV), a major cause of acute gastrointestinal disease, is a significant contributor to morbidity in the vulnerable, particularly in the immunocompromised and people at the extremes of age [1]. Norovirus became the most common cause of viral gastroenteritis after the introduction of the rotavirus vaccine in 2004 [2] and is responsible for over 685 million cases a year [3]. The norovirus genus in the *Caliciviridae* family is composed of at least ten genogroups (GI-GX) that are further divided into genotypes. Genogroups I and II are the predominant cause of acute norovirus gastroenteritis in humans with genogroup II, genotype 4 (GII.4) viruses causing the majority of human infections worldwide [4,5].

Noroviruses constitute a heavy medical and socioeconomic burden, with an estimated global annual impact of USD 60 billion [6]. Despite being a major cause of gastroenteritis [2,7,8], there are no approved antiviral medicines or vaccines available for the treatment or prevention of norovirus infections. As a result, there exists a strong need for the development of effective anti-noroviral therapeutics [3,9], with the HuNoV protease (EC 3.4.22) as a promising drug target [10,11,12].

The ~7.5 kb HuNoV genome contains three open reading frames (ORF), with the first ORF encoding the non-structural proteins designated NS1 to NS7, including the protease (Pro, NS6). The protease is responsible for processing the polyprotein into mature protein products and is essential for viral replication [13,14]. The 3C-like protease is the only protease encoded on the norovirus genome and is active as either a mature protease or as a precursor protein in association with the norovirus polymerase, both versions of which are active proteases, albeit with varying efficiencies [15].

The HuNoV protease has the general topology of a chymotrypsin-like protease with each monomer containing two domains (reviewed in [12]). The smaller N-terminal domain (Domain I, residues 1-73) is composed of a short alpha helix, followed by five antiparallel beta-strands (named βaI to βeI) that form a twisted antiparallel beta sheet. The larger C-terminal domain (Domain II, residues 74–181) has six antiparallel beta-strands (named βaII to βfII) that form an antiparallel beta-barrel. The two domains are connected by a long loop (residues 61–79).

The active site is formed from a cleft between Domains I and II, and three substrate-binding pockets (subpockets I, II, and IV) coordinate the positioning of the peptide substrate for nucleophilic attack. The catalytic cysteine residue Cys139 is in Domain II on a long loop between βcII-βdII. The catalytic residues His30 and Glu54 are in Domain I on loops between βcI-βdI and βdI-βeI, respectively [16]. Residues that interact with the substrate are mostly located within Domain II.

In the consensus mechanism of norovirus cysteine proteases, proteolytic catalysis occurs with Cys139 acting as a nucleophile. Nearby His30 is involved in the deprotonation of the cysteine to form a thiolate nucleophile, while Glu54 helps stabilise the protonated His30. The scissile peptide bond is cleaved via a covalent thioester intermediate.

Several small molecule inhibitors targeting the proteases of genogroup I (GI) noroviruses have been described, such as the site-directed peptide inhibitor MAP-I [16]. The analysis of a MAP-I-bound GI protease provided valuable information about substrate recognition and binding groups and validated rational structure-aided peptide design for anti-protease drug development [16]. Many other examples of inhibitor-bound GI HuNoV protease structures have also been determined, such as those described in references [17,18,19], with 51 crystal structures of GI noroviruses with and without inhibitors bound currently in the Protein Data Bank (PDB). In contrast, there are only two crystal structures of GII.4 proteases available (PDB codes 6NIR and 6B6I) which are both apoenzymes. This is despite GII.4 noroviruses causing 70–80% of all norovirus outbreaks in humans in the last decade [20].

The substrate binding pockets between GI and GII.4 proteases are structurally distinct by virtue of some key changes, notably a helix from residues 122 to 134 of the S1 pocket of the GII.4 protease, which is generally unstructured in GI pro, and a residue replacement at position 115 from His to Gly which results in the increased flexibility of a key loop in the S2 pocket [12]. As such, the strategy of optimising inhibitors for the GII.4 protease based on work carried out with the GI protease may not yield potent inhibitors [12]. The sequence identity within the GII.4 norovirus protease strains generally exceeds 95%, whereas the percent identity between GI and GII norovirus proteases’ amino acid sequences is only around 65% [21]. The 3D structures of HuNoV GII.4 proteases from the Minerva and Houston strains were solved in 2019 [21,22], revealing important conformational changes in the active site as compared to GI proteases. Although these structures have greatly improved our understanding of GII.4 proteases, an inhibitor-bound crystal structure of a HuNoV GII.4 protease has yet to be reported, which would further complement structure-directed inhibitor design.

**Figure 1:**
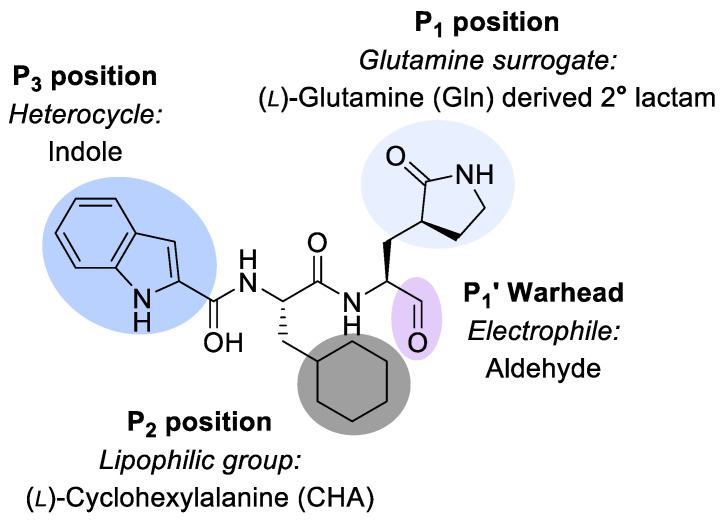
NV-004 structure. NV-004 contains an aldehyde as its electrophilic warhead (P1′, lilac), as well as a glutamine analogue (light grey, P1), a lipophilic group (dark grey, P2), and an indole heterocyclic group (blue, P3).

Compound NV-004 (Figure 1) has previously been reported as a protease inhibitor in RNA viruses [23]. It was initially designed to bind to the main protease (M^pro^) of SARS-CoV-2, which is also a 3CL-protease, appearing as **11a** in Dai et al., 2020 [23]. Its design is based on a peptide backbone, with chemical groups as side chains that complement subsites in the targeted protease (Figure 1). Binding orients an electrophilic “warhead” near the catalytic cysteine. NV-004 contains an aldehyde as its electrophilic warhead (P1′), as well as a glutamine analogue (P1), a lipophilic group (P2), and a heterocyclic group (P3).

Information on the interaction of effective inhibitors bound to GII.4 proteases is limited, despite the GII.4 noroviruses being the predominant genotype causing disease in humans. In this study, we report the 2.79 Å unliganded structure of the HuNoV GII.4 Sydney 2012 protease, confirm its inhibition by the inhibitor NV-004, and report the 1.83 Å GII.4 Sydney 2012 protease structure bound with NV-004.

## 2. Materials and Methods

### 2.1. Chemical Synthesis of Ligand NV-004

**Figure 2:**
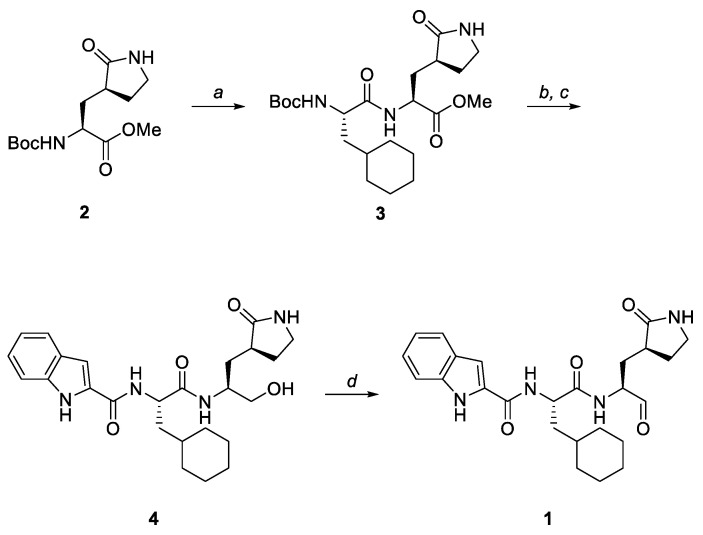
The synthetic scheme for NV-004 production. Reagents and conditions: (a) (i) 4 M HCl/1,4-dioxane, 0 °C to room temperature (rt), 1 h; (ii) Boc-Cha-OH, HCTU, NMM, DMF, 0 °C, 1 h, 86%; (b) (i) 4 M HCl/1,4-dioxane, 0 °C to rt, 1 h; (ii) indole-2-carboxylic acid, HCTU, NMM, DMF, 0 °C, 1.5 h; (c) NaBH_4_, MeOH, rt, 6.5 h, 63% over 2 steps; (d) Dess–Martin periodinane, CH_2_Cl_2_, 0 °C to rt, 2 h, 47%.

**Figure 3:**
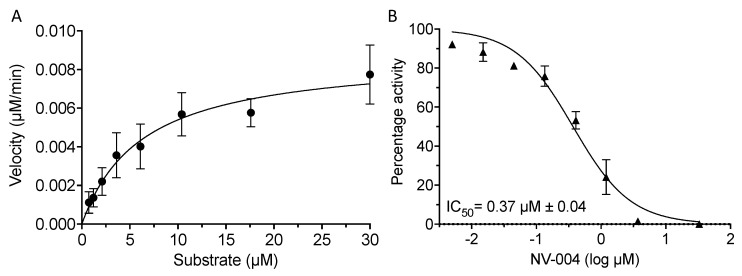
Kinetics and dose response curve of NV-004 with GII.4 Sydney 2012 Pro. (**A**) GII.4 Pro proteins (0.5 μM) were incubated with substrate and the reaction velocity as a function of substrate concentration (0.73–30 μM) was plotted. Kinetic values of 6.3 ± 3.3 μM, 2.9 × 10^−4^ ± 0.3 × 10^−4^ s^−1^, and 46.4 M^−1^ s^−1^ were determined for the K_m_, k_cat_, and k_cat_/K_m_, respectively. (**B**) GII.4 Pro at 0.5 μM was incubated with NV-004 for 40 min, following which, FRET peptide substrate (4 μM) was added. Normalised protease activity was plotted against log inhibitor concentration and the IC_50_ (μM) was calculated with GraphPad Prism software. Data represent the mean and standard deviation of three biological repeats.

**Figure 4:**
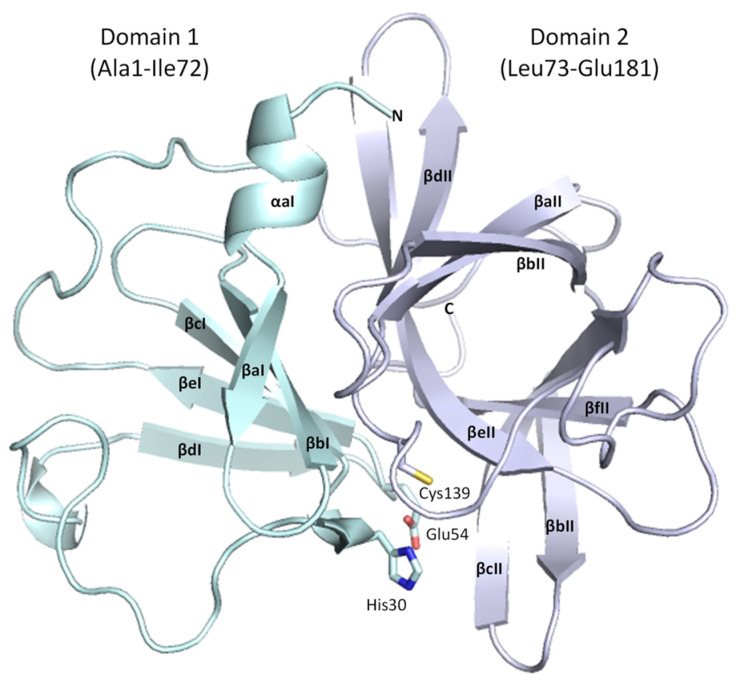
Overall structure of GII.4 HuNoV Sydney 2012 protease. Domain I is in cyan, and Domain II is in mauve. Alpha helices and beta strands are labelled consistent with the standardised nomenclature for norovirus proteases. This figure depicts Chain B from the ligand-free GII.4 HuNoV Sydney 2012 protease structure in PyMOL.

NV-004 (**1**) was synthesised following the scheme depicted in Figure 2 using a similar method to that previously reported by Dai et al. [23]. Ligand NV-004 (Figure 2, **1**) was synthesised according to the scheme in Figure 2. The *N*^α^-Boc protecting group of glutamine surrogate (Figure 2, **2**) was removed via treatment with HCl in 1,4-dioxane. Coupling of the resulting crude hydrochloride salt with Boc-Cha-OH was accomplished using HCTU and NMM in DMF to give dipeptide (Figure 2, **3**). The same conditions were then used for the removal of the *N*^α^-Boc protecting group of **3** and subsequent coupling with indole-2-carboxylic acid. The *C*-terminal methyl ester was then reduced using sodium borohydride to give alcohol (Figure 2, **4**). Finally, the oxidation of **4** using Dess–Martin periodinane afforded the desired aldehyde (Figure 2, **1**) in moderate yield. Further details on the synthesis of the ligand can be found in the Supplementary Methods and Materials. Spectroscopic data were consistent with literature values [23,24].

### 2.2. Chemical Synthesis of Fluorescent Protease Substrate

The fluorescent protease substrate 5(6)-carboxyfluorescein (FAM)-LGDYELQGPEDLAK-Dabcyl was synthesised using Fmoc solid phase peptide synthesis using an Fmoc-Rink amide linker attached to aminomethyl polystyrene resin (see Supplementary Methods and Materials).

### 2.3. Protein Expression and Purification

The HuNoV Sydney 2012 protease gene (Norovirus Hu/GII.4/Sydney/NSW0514/2012/AU, Genbank accession number: JX459908.1, base pairs 3029-3571) was cloned into a pRham^TM^ N-HisSUMO Kan vector (Lucigen, Middleton, WI, USA) containing an N-terminal His_6_ tag and SUMO fusion protein. The vector was transformed into *E. coli* cells (E.cloni, Lucigen) grown in LB medium supplemented with 12.5 μg/mL kanamycin to an OD_600_ of 0.8. Protein expression was induced with 0.2% rhamnose and incubated for four hours at 37 °C. Cells were harvested and lysed via sonication, and the supernatant was collected for purification. GII.4 Pro was purified from the supernatant by the use of Ni-NTA nickel agarose (Qiagen, Hilden, Germany). His_6_-SUMO-protease bound to the resin was placed on a gravity column at 4 °C and washed in 15 mM imidazole, pH 8. Protein was eluted in 150 mM imidazole, 50 mM HEPES, 150 mM NaCl, 10% glycerol pH 8 for enzymatic assays or 300 mM imidazole, 50 mM Tris, 150 mM NaCl, pH 8 for crystallography. The sample was dialysed overnight into either 50 mM HEPES, 150 mM NaCl, 10% glycerol, pH 8 or into 50 mM Tris, 150 mM NaCl, pH 8 to remove the imidazole. The SUMO-tag was cleaved using SUMO Express Protease (Lucigen) and removed from solution by Ni-NTA nickel resin. For enzymatic assays, cleaved and purified protein was dialysed into 50 mM HEPES pH 8, 150 mM NaCl and 50% glycerol and stored at −80 °C. For crystallography, the protein sample was dialysed into gel filtration buffer (20 mM MES pH 6, 150 mM NaCl, 5 mM DTT, 5% glycerol), concentrated to 5 mg/mL, and passed through a Superose 12 10/300 GL gel filtration column (Cytiva, Vancouver, BC, Canada) using an AKTA Purifier FPLC System (Cytiva). The central peak fractions of the elution peak were pooled and dialysed into 20 mM MES pH 6, 20 mM NaCl, 5 mM DTT, 5% glycerol. The sample was concentrated to 3 mg/mL.

### 2.4. Enzyme Kinetics and Inhibitor Activity Assays vs. Norovirus Protease

A fluorescence resonance energy transfer (FRET) assay was used to measure the enzyme activity of the Sydney 2012 GII.4 protease [25]. The peptide substrate, 5(6)-carboxyfluorescein (FAM)-LGDYEL**QG**PEDLAK-Dabcyl, was designed based on the canonical NS1/2-NS3 cleavage sequence with the cleavage site depicted in bold. Substrate was stored as a lyophilised compound until resuspension in 100% DMSO.

To establish the kinetic parameters, GII.4 HuNoV protease was diluted in assay buffer (10 mM HEPES pH 8, 30% glycerol, 0.1% CHAPS, and 10 mM DTT) to 0.5 μM and incubated with serial dilutions of FRET peptide substrate. The fluorescence was measured every minute at 492 nm (excitation) and 592 nm (emission) for 60 min at 37 °C. The background fluorescence of the respective substrate-only control was subtracted from fluorescence values with protease and corrected for inner filter effects. The corrected RFU was converted to the amount of product using a FAM standard curve and plotted over time. The initial rates of enzyme velocity were calculated by simple linear regression using the first 10 minutes of the progress curve. Velocity was plotted against the substrate concentration and the data were fit with the Michaelis–Menten equation to generate K_m_, k_cat_, and k_cat_/K_m_ values using GraphPad Prism Version 9 (GraphPad Software, Boston, MA, USA).

For IC_50_ determination, the GII.4 HuNoV protease was diluted in assay buffer to a final concentration of 0.5 µM and incubated with increasing concentrations (0.015–33 µM) of NV-004 in black 96-well plates (Greiner, Pleidelsheim, Germany) for 40 min. FRET peptide substrate was added to reaction wells at a final concentration of 4 μM and mixed for two minutes at 700 revolutions per minute (rpm). Reactions were incubated at 37 °C and fluorescence was monitored every minute at 492 nm (excitation) and 592 nm (emission) for 60 min on a VICTOR Nivo microplate reader (Perkin Elmer, Hamburg, Germany). To calculate the IC_50_, the background fluorescence was subtracted, and the initial rates were derived from the first 10 min of the progress curves. The initial rates were normalised and plotted against the NV-004 concentration, and the IC_50_ was calculated using non-linear regression software (log inhibitor (x-axis) vs. normalised response (y-axis)) in GraphPad Prism Version 9.

### 2.5. Crystallisation

All crystals were grown in sitting drops using the Hampton PEG/ION™ screen (Hampton Research, Aliso Viejo, CA, USA). Drops of 300 nL total volume (150 nL of protein/150 nL of screen) were set using a Mosquito crystallisation robot (SPT Labtech, Melbourne, UK) and incubated at 16 °C in a Rock Imager (Formulatrix, Bedford, MA, USA). Unliganded protease crystals grew in 3 days in 20% *w*/*v* PEG 3350, 8% Tacsimate pH 5. The protein buffer contained 20 mM MES pH 6, 20 mM NaCl 5 mM DTT, and 5% glycerol.

Crystals of the protease-NV-004 complex were grown by first incubating purified GII.4 HuNoV protease (at 3 mg/mL in a buffer containing 20 mM MES pH 6, 20 mM NaCl 5 mM DTT, and 5% glycerol) with 1 mM of NV-004 (dissolved in 100% DMSO and a final concentration of 10% DMSO) for 45 minutes on ice. This is an approximate 6-fold molar excess of inhibitor. The protein-inhibitor complex was centrifuged for 20 min at 16,000× *g* in a benchtop centrifuge (Eppendorf Microcentrifuge 5415 R), then utilised for crystallisation trials and experiments. Co-crystals nucleated after two months in a condition containing 20% *w*/*v* PEG 3350, 8% Tacsimate pH 4. Crystals were harvested, transferred into a cryoprotectant containing mother liquor and 15% (*v*/*v*) glycerol, and flash frozen in liquid nitrogen. Diffraction data were collected at the Australian Synchrotron at the ANTSO Research Facility on the Macromolecular Crystallography (MX2) beamline [26].

### 2.6. Data Collection, Structural Determination and Refinement

XDS [27] and Aimless [28] were used for data processing. Data from the Sydney 2012 unliganded protease crystal were processed in the C222_1_ space group with four molecules per asymmetric unit. NV-004-bound protease was processed in C2 with one chain in the asymmetric unit. Phases were solved using molecular replacement using PHASER [29] within PHENIX [30]. The previously solved GII.4 Minerva protease (PDB ID: 6B6I, percent identity; 96.7%) was used as the initial search model. Following automated model building using PHENIX autobuild, multiple iterations of building and refinement were carried out using COOT [31] and phenix.refine, respectively. Geometric restraints for NV-004 were generated in eLBOW [32] using the SMILES string: O=C[C@H](C[C@H](CCN1)C1=O)NC([C@H](CC1CCCCC1)NC(c1cc(cccc2)c2[nH]1)=O)=O. A covalent bond was introduced between the ligand and Cys139 of the protease via the custom geometry restraint feature in PHENIX. Occupancies for the NV-004 ligands were refined in PHENIX. Hydrogen bonds, covalent bonds, and hydrophobic interactions were calculated in LigPLOT PDBSum [33]. Overall structure validation was performed in MolProbity [34] and wwPDB validation service [33], and ligand validation was performed via Polder map analysis [35]. Protein visualisation was performed in UCSF ChimeraX [36]. The isotropic B-factors were calculated using PHENIX. The real-space correlation factor was calculated in CCP4i using Procheck [37]. RMSDs were calculated using the align function in PyMOL (Schrödinger LLC, New York, NY, USA) using all protein atoms.

Data collection for the ligand-free data set consisted of 1800 frames at 0.1°/frame collected at 1°/sec with a detector distance of 300 mm, while data collection for the NV-004 bound structure consisted of 2400 frames at 0.1°/frame collected at 1°/sec with a detector distance of 270 mm.

### 2.7. Accession Number for Protein Structures

The crystal structure coordinates and structure factors for the ligand-free and NV-004-bound protease structures have been deposited in the Protein Data Bank under accession numbers 8U1V and 8U1W, respectively (https://www.rcsb.org, accessed on 25 September 2023). Data collection and refinement statistics are in Table 1, and sample diffraction images are in Supplemental Figure S1.

## 3. Results

### 3.1. Inhibitor Activity Analysis

GII.4 Pro showed a K_m_ of 6.3 μM (Figure 3A) when assayed with three separate preparations of protein, and in the subsequent IC_50_ analysis with NV-004, the substrate concentration was maintained below K_m_ at 4 μM. A titration of NV-004 in the presence of GII.4 Pro produced an IC_50_ of 0.37 μM (±0.04 μM) (Figure 3B). The efficacy of this inhibitor prompted structural studies on the complex of NV-004 with the GII.4 protease.

### 3.2. Structure of the GII.4 Sydney 2012 HuNoV Protease in the Ligand-Free State

The GII.4 Sydney 2012 unliganded protease crystallised in space group C222_1_ with four chains in the asymmetric unit. The electron density quality was consistent with a 2.79 Å structure with continuous density for each protein chain, some missing residues at the N- and C-terminus, and with several side chains omitted from the model due to weak density. The average overall isotropic B-factor was 65.2 Å^2^ and the electron density had an average real-space correlation factor of 0.884. The average RMSD between chains was 1.35 Å for the complete chain, 1.03 Å for Domain I, and 1.51 Å for Domain II. A full list of RMSD values can be found in the supplemental section (Table S1).

The structure of the ligand-free HuNoV protease shares conserved architecture with other GII.4 3C-like proteases [21,22], possessing a chymotrypsin-like fold, with the active site located between the N-terminal five-stranded β-sheet domain (Domain I, residues 1–72, Figure 4) and the C-terminal six-stranded antiparallel β-barrel domain (Domain II, residues 73-181, Figure 4). The His30 and Glu54 residues of the catalytic triad are contained within Domain I, whilst the catalytic cysteine nucleophile (Cys139) is contained within Domain II.

#### Conformation of Arg112

In the ligand-free protease crystal structure, we observed two distinct conformations for Arg112 in the Sydney 2012 GII.4 protease. Based on prior structural studies, it has been suggested that Arg112 adopts a position that is important for the functioning of the catalytic triad [21,22]. In three chains (Chains B, C, D), the guanidino group of Arg112 is oriented towards the active site and interacts with Glu54. As noted in earlier reports, Arg112 in this closed conformation likely prevents the unprotonated imidazole nitrogen of His30 from being positioned optimally to deprotonate Cys139 for nucleophilic attack [21]. Arg112 also occupies the middle of the active site cleft and may sterically hinder the entrance of substrate.

**Figure 5:**
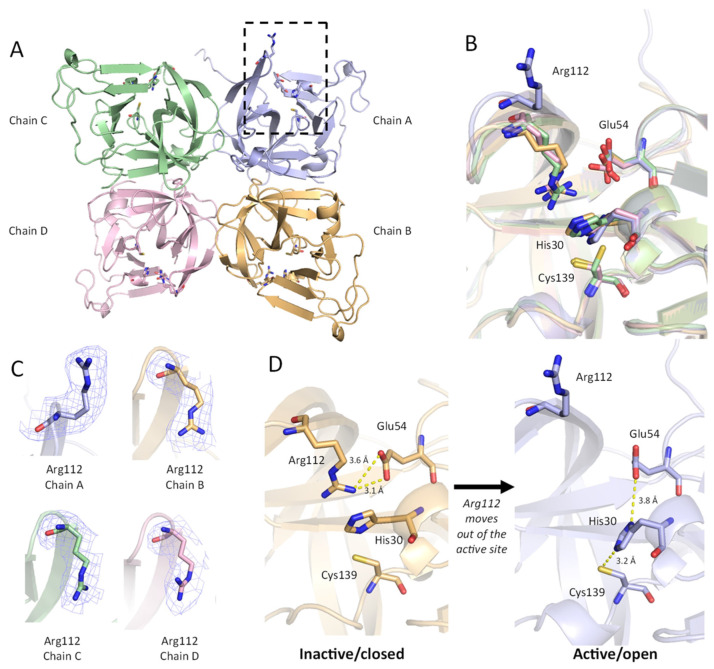
Conformation of Arg112 affects the active site residues. (**A**) The asymmetric unit of GII.4 HuNoV protease (ligand-free) is shown as a protein cartoon. Chain A is purple, Chain B is orange, Chain C is green, Chain D is pink. Residues His30, Glu54, Arg112, and Cys139 are shown as sticks. The active site and Arg112 are indicated in a dashed box for Chain A with the active site open. (**B**) Overlay of the four chains in the ASU, showing a distinct conformation for Arg112 in Chain A. (**C**) Polder maps (3.0 σ) for Arg112 in Chains A–D. (**D**) Changes in active site conformation between Chain B (inactive/closed) and Chain A (active/open). Polar interactions between Arg112 and active site residues His30, Glu54, and Cys139 are indicated with dashed yellow lines.

In contrast to the closed conformation observed in chains B–D, one chain (chain A) adopts an alternative conformation, whereby Arg112 does not interact with the catalytic triad but faces away from the catalytic centre (Figure 5A,B). In this orientation, the Arg 112 side chain is involved in a crystal contact that includes residues Ala127, Lys128, Ser129, and Thr134 from an adjacent chain (Supplemental Figure S2). The positioning of Arg112 in this open conformation was validated with Polder maps (Figure 5C) [35]. The movement of Arg112 out of the active site makes Glu54 more available to interact with His30, which is oriented toward Cys139 for deprotonation, promoting an active state (Figure 5D).

### 3.3. Structure of the GII.4 Sydney 2012 HuNoV Protease Bound to Inhibitor NV-004

The GII.4 HuNoV protease, when complexed with the inhibitor, NV-004, produces crystals in space group C2 that diffract to 1.8 Å. This crystal form has one chain in the asymmetric unit. The average overall isotropic B-factor is 30.2 Å^2^ and the electron density has an average real-space correlation factor of 0.950. Electron density is generally excellent throughout the structure, with a few exceptions. There is a main chain break of eight residues in the loop between Thr123 and Asp131; this loop region is commonly disordered in inhibitor-bound norovirus protease structures but is often well-ordered in ligand-free enzyme structures [17,38,39,40,41]. Residues 163–164 also have weak density and were not included in the model. Residues 173–181 at the C-terminus and seven side chains with weak density were also omitted.

**Figure 6:**
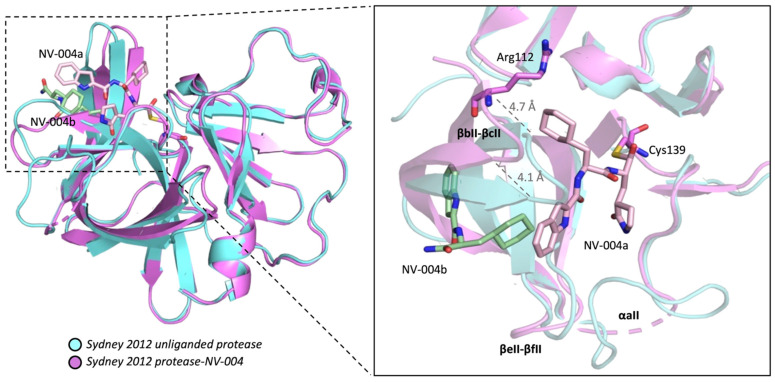
GII.4 HuNoV Sydney 2012 protease with bound inhibitor NV-004. Protease-NV-004 is shown in ribbon representation (hot pink) superposed on the unliganded protease (blue). NV-004a (pink, site 1) in the active site and NV-004b (green, site 2) in the S4 pocket are displayed in stick representation. Distances were measured in USCF ChimeraX. NV-004 molecules are shown as sticks with oxygen atoms in red and nitrogen atoms in blue. Active site Cys139 side chain shown as a stick. Structural elements are labelled in bold.

**Figure 7:**
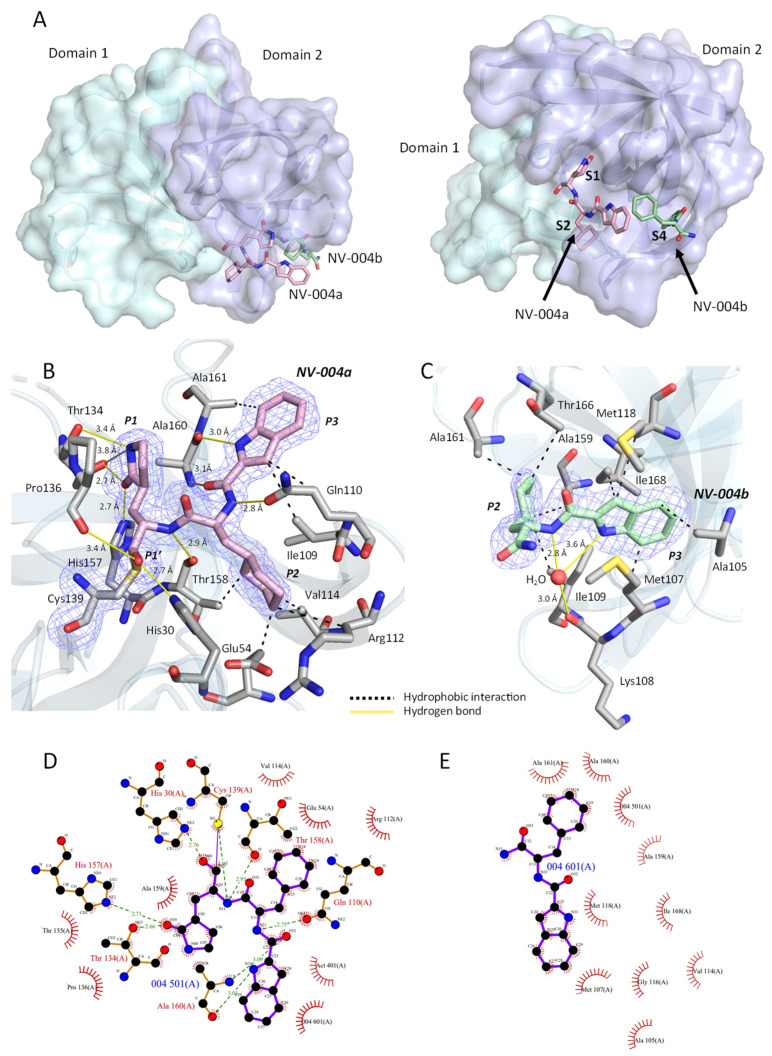
NV-004 bound to GII.4 HuNoV Sydney 2012 protease. (**A**) Location of NV-004 bound to GII.4 HuNoV Protease. The protease is shown in two different orientations. In each orientation, one protomer of the protease is displayed as a surface representation coloured by the domain. Domain I is in cyan; Domain II is in purple. The two NV-004 molecules are shown as sticks. NV-004a (pink) is located at the active site (S1–S2), and NV-004b (green) is in a nearby site (S4). Subpockets are labelled in bold. (**B**) Polder map density (contoured to 3σ) for NV-004a is shown as a blue mesh. NV-004a (pink sticks) is in the S1/S2 subsite. Residues of protease that interact with NV-004a are shown as sticks (grey). Hydrogen bonds are shown as solid yellow lines. Hydrophobic interactions are shown as dashed black lines. (**C**). Polder map density (contoured to 3σ) for NV-004b is shown as a blue mesh. NV-004b (green sticks) is found in the S4 subsite. Residues of protease that interact with NV-004b are shown as sticks (grey). A water molecule that forms a water-mediated H-bond with NV-004b and the protease is shown as a red sphere. (**D**). LigPLOT diagram for NV-004a. (**E**) LigPLOT diagram for NV-004b displaying the bonds involved in the interactions between the NV-004 and GII.4 HuNoV protease. Green lines denote hydrogen bonds, with distances shown in Å; the thin purple line denotes the covalent bond between NV-004 and the protein. Red radials represent non-bonded hydrophobic interactions.

Structural alignments between the Sydney 2012 ligand-free protease and the NV-004-bound structure reveal high structural conservation within Domain I (average RMSD 1.059 Å), and somewhat more structural variation within Domain II (average RMSD 1.675 Å). A full list of RMSD values can be found in the supplemental section (Table S2). In Domain II, structural discrepancies were observed in three regions located close to the active site: the loop between βbII-βcII containing His104-Gln117, the loop between βeII-βfII containing Ala161-Asn165, and αaII containing Thr123-Leu132 (which is disordered in the protease-NV-004 structure). In the protease-NV-004 structure, the βbII-βcII strands shift 4.7 Å (at the farthest distance) from the relative position of βbII-βcII in the unliganded protease structure (Figure 6). This places Gln110 within hydrogen bonding distance (2.75 Å) with the NH of the P2 ring of NV-004a (as shown later in Figure 7B). Further, the βeII-βfII loop shifts 6.4 Å with respect to the relative position of the loop in the ligand-free enzyme. Finally, αaII could not be modelled in the protease-NV-004 structure due to discontinuous electron density, although αaII is well ordered in all chains of the Sydney 2012 unliganded enzyme.

#### Two Binding Sites for NV-004

Prior to modelling any bound ligands, well-defined positive electron density was observed in the F_o_-F_c_ maps (Supplemental Figure S3) in two different pockets of the protease, termed the S1–S2 and S4 subpockets. These pockets are a part of the cleft between domains I and II. The density at both sites was consistent with bound NV-004 molecules and is described below.

- NV-004 site 1

The strongest density was located at the anticipated ligand binding site at S1–S2, and the size and shape of this density was consistent with the full chemical structure of NV-004. This ligand was built in its entirety at this location (termed NV-004a, Figure 7B). Densities for the P1′, P1, P2, and P3 groups were well defined, and the ligand refined well within the density. There was strong continuous density consistent with a covalent bond between the S atom of Cys139 and the C atom of the aldehyde (P1′) of NV-004a.

As well as being covalently bound to Cys139, NV-004a forms eight hydrogen bonds with the protease. Hydrogen bonding groups of NV-004a are on the P1, P1′, and P3 groups, as well as the peptide bonds between them. As depicted in the protease active site (Figure 7B), the O atom of the P1 group H-bonds with the side chain of His157 and the side chain of Thr134, each with a distance 2.7 Å, and the N atom of the P1 side chain forms hydrogen bonds with the backbone carbonyl and side chain of Thr134, with distances of 3.4 and 3.8 Å, respectively. The P3 side chain N atom forms a hydrogen bond with the carbonyl group of Ala160 with a distance of 3.0 Å. The O atom of P1′ forms a hydrogen bond with the N atom of His30 with a distance of 2.7 Å and with the carbonyl of Pro136, with a distance of 3.4 Å. There are also hydrogen bonds formed between the peptide bonds in NV-004a and the protease. The NH of the peptide bond between P2 and P3 hydrogen bonds with the side chain of Gln110 at 2.8 Å. The carbonyl of the peptide bond between P2 and P3 forms a hydrogen bond with the backbone NH of Ala160 at 3.1 Å. Finally, the NH of the peptide bond between P1 and P2 hydrogen bonds with the carbonyl of Thr158 with a distance of 2.9 Å. The P2 side group forms no hydrogen bonds but has hydrophobic interactions with surrounding residues Glu54, Arg112, Val114, and Thr158. The interactions between NV-004a and the GII.4 protease were also calculated via LigPLOT (Figure 7D), with the exception of the longer H-bonds between the N atom of P1 and Thr134, which have distances that exceed the cutoff for H-bond prediction in LigPLOT.

- NV-004 site 2

NV-004b was modelled into unexpected electron density that was identified within the S4 subpocket (Figure 7A). The density found at this site closely resembled the P3 and P2 groups of NV-004 but lacked any density resembling its P1′ and P1 moiety. LigPLOT analysis indicates interactions between NV-004b and the protease are due to hydrophobic contacts with Ala105, Met107, Ile109, Met118, Ala159, Ala161, Thr166, and Ile168, as well as with the P3 group of NV-004a (Figure 7C,E). In contrast to NV-004a, no direct hydrogen bonds or covalent interactions were observed between NV-004b and the protease. However, a water-mediated hydrogen bond formed with the backbone carbonyl of Lys108, as well as two nitrogen atoms in NV-004b, and as it was the only polar contact observed in this site (Figure 7C), it may be important in orienting the NV-004b molecule in the S4 site.

Over the course of building the NV-004 bound structure, we observed a drop in R_work_ and R_free_. Prior to adding the two ligands NV-004a and NV-004b to the model, the R_work_ was 0.194 and R_free_ was 0.229. The inclusion of NV-004 into the protease model at both the S1/S2 and S4 sites resulted in an R_work_ of 0.165 and R_free_ of 0.191. The drop in R_work_ and R_free_ confirmed the inclusion of the ligands improved the model. After refinement was completed, both molecules of NV-004 were validated by polder maps (Figure 7B,C) and analysed by LigPLOT within PDBsum (Figure 7D,E).

## 4. Discussion

The norovirus 3C-like protease is functionally indispensable for virus replication, and therefore represents an attractive biological target for anti-protease inhibitor design. Such work is aided by the availability of 3D atomic structures, against which existing drugs can be modified and new drugs developed. Whilst more than 50 GI.1 protease structures have been reported in the PDB, only two structures are available for GII.4 proteases, and no inhibitor-bound GII.4 protease structures have been reported. To date, only ligand-free structures have been used in molecular docking simulations to predict potential inhibitor binding and assist in anti-GII.4 inhibitor design [22,42,43]. This study contributes an additional two GII.4 protease structures to the field, unliganded GII.4 Sydney 2012 protease and the NV-004-bound form.

Within the unliganded GII.4 protease structure, both the active and inactive conformations of Arg112 were observed and are available to support structure-guided drug development efforts. The unliganded protease was solved with one chain in the open conformation and three in the closed, indicating that there is enough flexibility in this region of the structure for the active conformation to be available for the subunits in the GII.4 Sydney 2012 protease. In one of the subunits, an extensive crystal contact was formed, which allowed the open form to be stabilised. This contrasts with work by Viskovska et al. [21], who found all chains of GII.4 protease to be in the closed conformation in the same pH buffer conditions (pH 6), indicating there may be more flexibility at this pH than originally thought. Regardless, within the unliganded Sydney 2012 GII.4 protease structure, both the active and inactive conformations were observed and will support structure-guided drug development efforts. Previous studies on the GII.4 Houston strain [21,22] have shown the conformation of this side chain alters the electrostatic potential of the active site (Supplemental Figure S4) and may play a role in regulating the proteolytic activity of the protease.

A second partial structure of NV-004 (NV-004b) was observed in the S4 pocket at high occupancy (Table 1), although density for the P1′ and P1 groups of the inhibitor was missing, potentially due to a lack of stabilising contacts between the protease and the groups at this site. LigPLOT analysis indicated that NV-004b is held in place only by non-bonded hydrophobic interactions. However, despite a lack of orienting salt bridges and hydrogen bonds, the structural order observed in the electron density map for the P3 heterocycle of NV-004b is greater than one would expect. To our knowledge, there are no inhibitors which utilise the S1-S3 binding pockets as their primary site and also bind to the S4 pocket as an independent additional site. Whether this interaction at the S4 pocket is inhibitory to the enzyme, influences the observed IC_50_ for NV-004, or affects the interaction mode of the inhibitor with the protease is unknown.

Previous work on peptidomimetics has shown a utility for the S4 pocket in norovirus drug design, with the S4 subpocket conserved among HuNoV proteases [12]. Indeed, all the substrate-binding pockets are involved in substrate recognition and in orienting the substrate for nucleophilic attack by the catalytic cysteine during polyprotein processing [12,44]. Some studies have exploited this feature in drug design. For example, some peptidomimetic inhibitors designed against the GI.1 proteases have hydrophobic groups which occupy the S4 pocket [10,17]. For example, protease inhibitors syc-10 and syc-59 were observed to have hydrophobic groups in the S4 pocket and make substantial van der Waals contacts through Ala160, Thr161, Thr166, Val168, Met107, and Ile109 [45]. NV-004b makes the same contacts, except for I109. A further study which docked a protease peptidomimetic (referred to as Compound 19) in a HuNoV GI.1 protease structure (PDB: 4XBB) predicted the naphthyl ring of the inhibitor to bind the S4 pocket and make contacts with Ile109, Thr161, and Leu162 [42].

This study presents the structure of an inhibitor bound in the active site of norovirus GII.4 proteases and shows how they might differ from GI proteases. A previous study modelled a similar compound but with a quinolone group in the P3 position instead of an indole [43]. While these compounds show marked differences in IC_50_ that may reflect alternate binding, comparing the modelled interaction to the inhibitor-bound structure detrmined in this study reveals significant differences in interactions within the active site.

We note that NV-004 was originally developed for the inhibition of the M^pro^ of SARS-CoV-2 (compound 11a in [23]). Here, we have presented an example of the successful repurposing of an anti-M^pro^ inhibitor. Indeed, the active sites of SARS-CoV-2 M^pro^ and HuNoV are structurally similar, and a recent comprehensive review has highlighted a library of anti-SARS-CoV and SARS-CoV-2 inhibitors [46]. This work may inspire further efforts to repurpose coronavirus inhibitors for their potential anti-norovirus effects.

In summary, the results from this study contribute two structures to the research field of the GII.4 noroviruses. The unliganded structure reveals the open and closed active site conformations of the GII.4 Sydney 2012 protease, which aligns with previous observations of other GII.4 proteases and will support anti-GII.4 protease inhibitor development. The high-resolution structure of GII.4 Pro in complex with NV-004 reveals the residue interactions involved in covalent and non-covalent binding of the inhibitor and supports further structure-guided drug improvements.

## Figures and Tables

**Table 1:** Data collection and refinement statistics for GII.4 HuNoV protease.

Data Collection	Ligand-Free	NV-004-Bound
Space group	C222_1_	C2
Cell dimensions		
a, b, c (Å)	112.8, 160.9, 95.3	79.4, 53.4, 50.0
α, β, γ (°)	90.0, 90.0, 90.0	90.0, 102.3, 90.0
Wavelength (Å)	0.95364	0.95364
Resolution range (Å)	2.79–48.54 (2.79–2.94) ^a^	1.84–48.85 (1.84–1.88)
Observations	143,659 (19,227)	82,094 (4830)
Unique reflections	21,735 (2936)	17,894 (1050)
*R*_merge_ (*I*)	0.156 (1.26)	0.037 (0.43)
*R*_meas_ (*I*)	0.184 (1.48)	0.047 (0.55)
*R*_pim_ (*I*)	0.097 (0.78)	0.029 (0.34)
Mean I/σ	7.1 (1.0)	14.2 (2.1)
Mean *CC*_1/2_	0.994 (0.512)	1.0 (0.85)
Completeness (%)	99.1 (93.9)	99.7 (97.4)
Multiplicity	6.6 (6.5)	4.6 (4.6)
Protein chains in ASU	4	1
No. of atoms		
Protein	5006	1203
Ligands (all)	n/a	79
NV004a	n/a	33
NV004b	n/a	23
Water	9	102
R_work_	0.194	0.165
R_free_	0.214	0.191
RMSD from ideal		
Bond length (Å)	0.002	0.006
Bond angle (°)	0.483	0.900
Ramachandran Plot		
Favoured (%)	96.8	98.7
Disfavoured (%)	3.2	2.3
Outliers (%)	0	0
Clash score (%-tile) ^b^	2.63 (100)	1.62 (100)
MolProbity score (%-tile) ^b^	1.25 (100)	0.91 (100)
B-factors (Å^2^)		
Average	65.2	30.2
Protein	n/a	29.5
Ligands (all)	n/a	41.4
NV004a	n/a	26.7
NV004b	n/a	37.4
Water	55.9	38.16
Occupancies (%)		
NV-004a	n/a	100
NV-004b	n/a	89
PDB ID	8U1V	8U1W

^a^ Values for the outer resolution shell of data are given in parenthesis. ^b^ From MolProbity.

## Data Availability

The crystal structure coordinates and structure factors for the ligand-free and NV-004-bound protease structures have been deposited in the Protein Data Bank under accession numbers 8U1V and 8U1W, respectively (https://www.rcsb.org).

## Supplementary Materials

The following supporting information can be downloaded at: https://www.mdpi.com/article/10.3390/v15112202/s1, Figure S1: Diffraction patterns; Figure S2: Arg112 of Chain A is stabilised in the out conformation by hydrogen bonding with a crystallographic symmetry mate; Figure S3: Modelling NV-004 binding to Sydney 2012 Pro; Figure S4: Structure of the Sydney 2012 GII.4 HuNoV protease; Table S1: RMSDs: Comparing the four chains in the asymmetric unit of the ligand-free protease structure; Table S2: RMSDs: NV-004-bound protease compared with each chain in the ligand-free protease structure; Supplementary Methods and Materials.
