# Supplementary material for: Crystal Structure of Inhibitor-Bound GII.4 Sydney 2012 Norovirus 3C-Like Protease
Source: Viruses. 2023 Oct 31;15(11):2202. doi: 10.3390/v15112202 (PMC10674469; doi:10.3390/v15112202)
Supplement: Supplementary file 1 [file viruses-15-02202-s001.zip › viruses-2660252-supplementary.pdf]

Supplemental Information, Eruera et al.

Crystal structure of inhibitor-bound GII.4 Sydney 2012 Norovirus 3C-like protease.

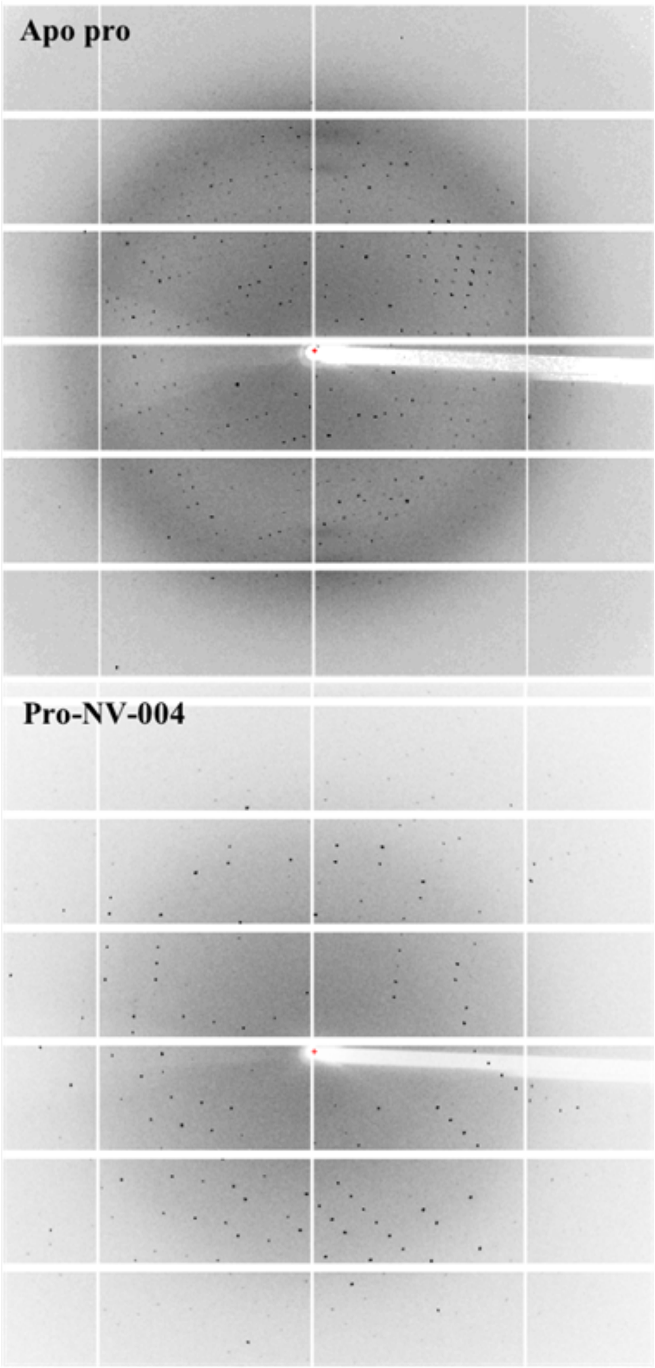

Figure S1. Diffraction patterns. Diffraction was analysed at the MX2 beamline at the Australian Synchrotron (ANTSO Research) in Melbourne, Australia. The ligand free HuNoV protease crystal (*top diffraction pattern*) was screened at beam energy of 13001.15 eV, detector distance of 300 mm, exposure time of 2 seconds, and a 10° wedge. The pro-NV-004 crystal (*bottom diffraction pattern*) was screened at beam energy of 13001.15 eV, detector distance of 270 mm, exposure time of 0.5 seconds, and a 2° wedge.

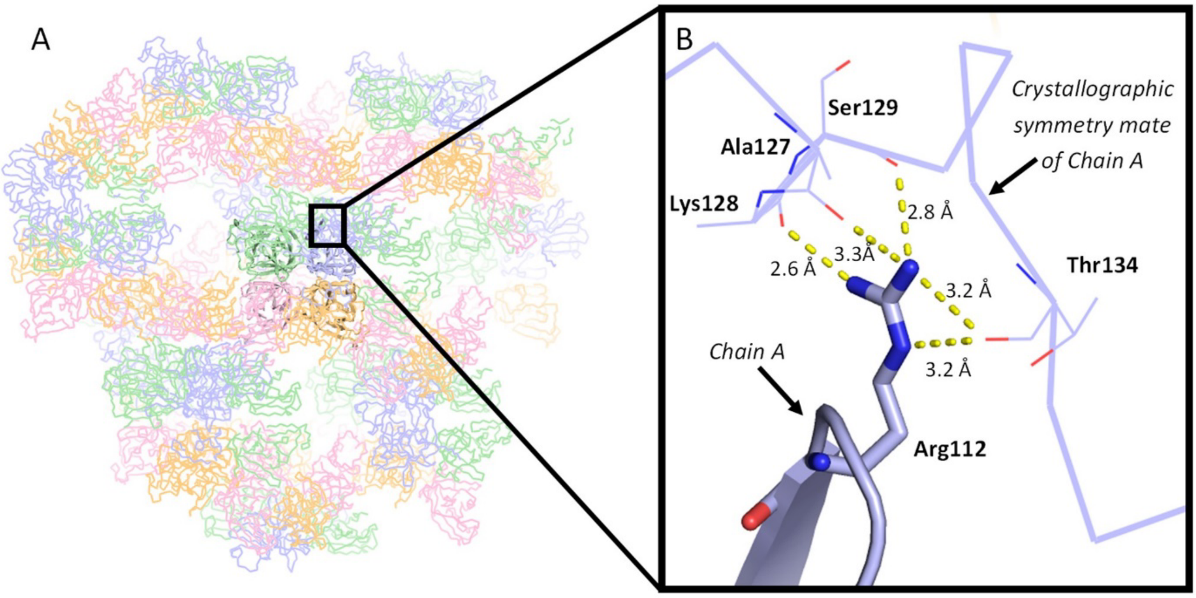

Figure S2. Arg112 of Chain A is stabilized in the open conformation by hydrogen bonding with a crystallographic symmetry mate. A) crystal packing in the ligand free structure. The four chains of the ASU are shown in the centre, represented by cartoons and coloured by chain. Chain A is purple, Chain B is orange, Chain C is green, Chain D is pink. Symmetry related ASUs are shown as ribbons using the same colouring. B) Zoom in on the interactions formed between Arg112 of Chain A and a symmetry related Chain A. Chain A of the central ASU is shown as cartoon, with Arg112 shown as sticks. The crystallographic symmetry mate of chain A is shown as ribbons, with Ala127, Lys128, Ser129 and Thr 134 shown as sticks. Residues 128-134 form two distinct conformations in the crystal structure, suggesting this is a flexible region of the protein. Chain C is similar to Chain A, and Chain B and D are similar to one another. The two loop conformations deviate by ~6 Å. Although residues 128-134 are in a similar conformation in chain A and C, this region does not make crystal contacts with Arg112 in chain C like it does for chain A. Hydrogen bonding interactions are shown as dashed yellow lines.

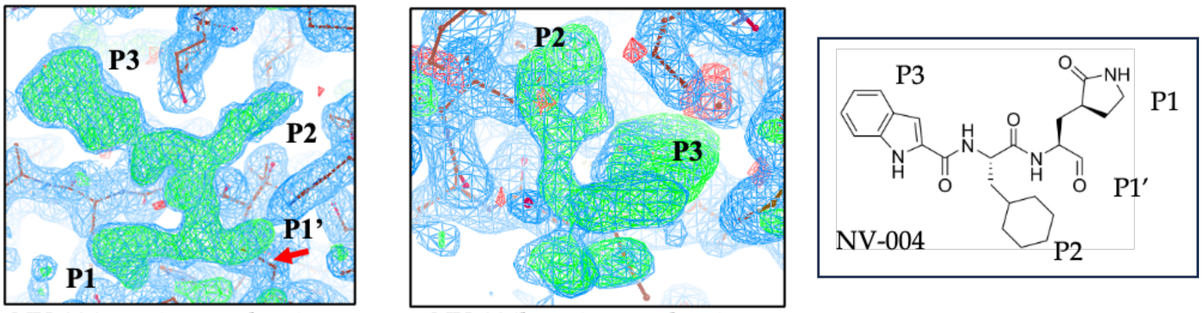

NV-004a omit map density

NV-004b omit map density

Figure S3. Modelling NV-004 binding to Sydney 2012 Pro. Unassigned electron density for two molecules of NV-004 were observed in the density map prior to the inclusion of the ligands in the model. Density in the F_o-F_c map was observed for a complete molecule of NV-004a and the covalent bond it forms with the catalytic cysteine (*red arrow*). Partial density corresponding to the P3 and P2 of NV-004b was present in the S4 subpocket.

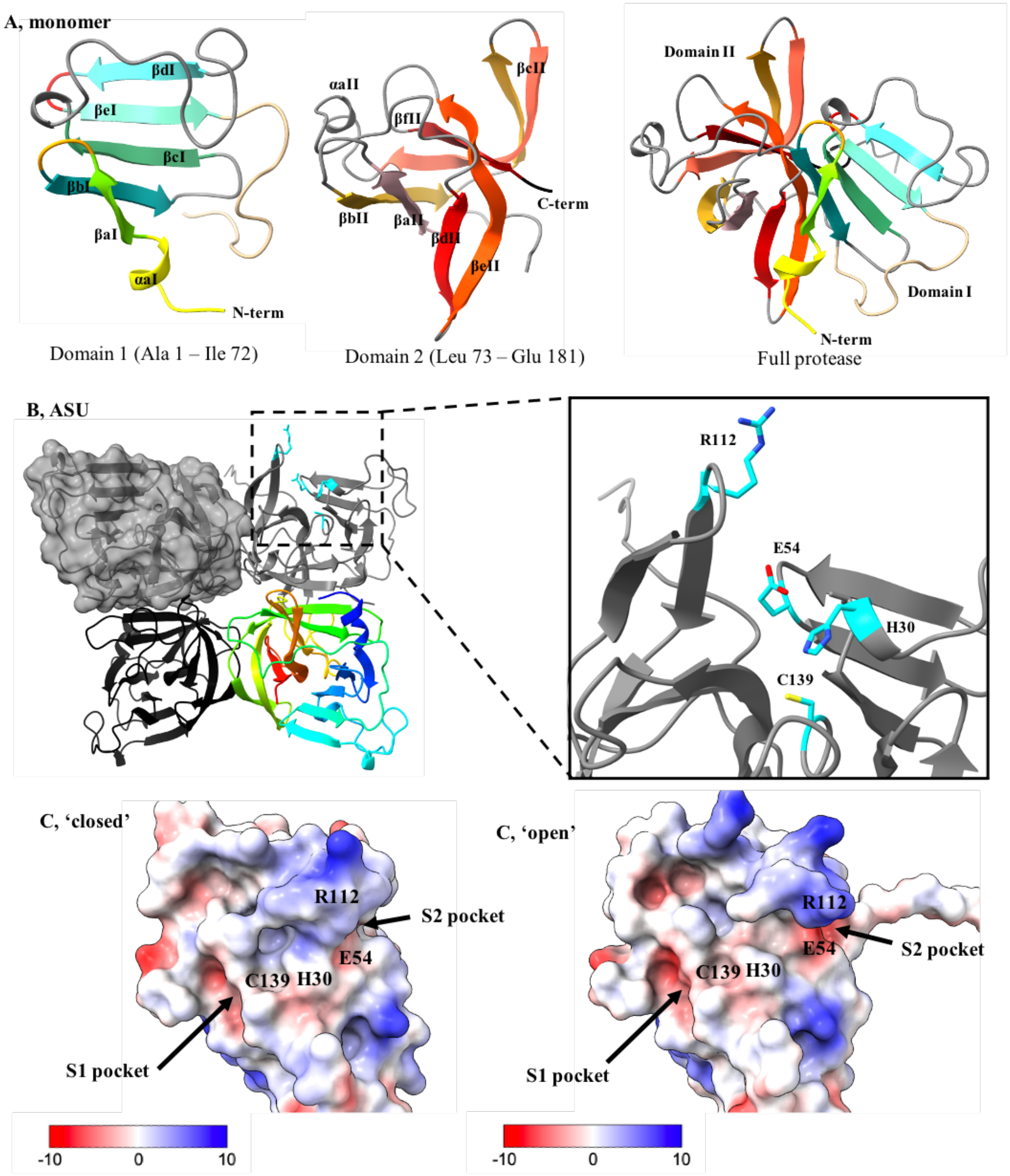

Figure S4. Structure of the Sydney 2012 GII.4 HuNoV protease. (A) A single monomer is shown segmented into Domains I and II, coloured from yellow (N-term) to red (C-term). The linker which connects the domains is coloured in beige. Protein fold names are labelled. A full monomer is displayed on the right. (B) The asymmetric unit of the unliganded protease crystal is shown with one chain covered in molecular surface. The sidechains of the catalytic triad and R112 of chain A are displayed in cyan. (C) An electrostatic potential map of the 'closed' and 'open' active sites of the protease reveals that the electrostatic potential of the active site is altered because of conformational changes in R112. The outward-pointing position of R112 in the 'open' conformation results in an increase in negative potential near the catalytic cysteine (C139) and the glutamic acid (E54). The catalytic triad and R112 residue are labeled, and S1 and S2 substrate-binding pockets are indicated by an arrow. Surface electrostatic potential is assigned red for negative, white for neutral and blue for positive potential using columbic colouring in ChimeraX. Electrostatic scale displayed in kT/*e* from -10 to 10. Note, the 'closed' chain (*chain A*) lacked density for modeling the C-terminal tail.

Table S1. Root-mean-square deviations (RMSDs) of atomic positions comparing the four chains in the asymmetric unit of the ligand-free protease structure. Each chain of the ligand-free protease structure is listed on the x- and y-axis of a matrix. The numbers within the matrix represent the measure of the average distance (Å) between the atoms of superimposed protein chains in the ligand-free protease structure. All protein atoms were included for the superposition and RMSD calculations. A) Overall RMSD was calculated using all residues (1-181). B) Domain I RMSD was calculated using residues 1-72. C) Domain II RMSD was calculated using residues 73-181.

A) Overall RMSD (Å)

Chain:	A	B	C	D
A		1.676	1.358	1.467
B	1.676		1.42	0.705
C	1.358	1.42		1.425
D	1.467	0.705	1.425	

Average: 1.353

B) Domain I RMSD (Å)

Chain:	A	B	C	D
A		1.21	0.848	1.081
B	1.21		1.051	0.76
C	0.848	1.051		1.184
D	1.081	0.76	1.184	

Average: 1.026

C) Domain II RMSD (Å)

Chain:	A	B	C	D
A		1.905	1.621	1.681
B	1.905		1.594	0.629
C	1.621	1.594		1.532
D	1.681	0.629	1.532	

Average: 1.493

Table S2. Root-mean-square deviations (RMSDs) of atomic positions comparing NV-004 bound protease to each chain in the ligand-free protease structure. Each chain of the ligand free structure is listed on the x-axis and the NV-004 bound structure is listed on the y-axis of a matrix. The numbers represent the measure of the average distance (Å) between the atoms of superimposed protein chains in the ligand-free protease structure compared with the NV-004 bound structure. All protein atoms were included for the superposition and RMSD calculations. A) Overall RMSD was calculated using all residues (1-181). B) Domain I RMSD was calculated using residues 1-72. C) Domain II RMSD was calculated using residues 73-181.

A) Overall RMSD (Å)

	Ligand-free			
	A	B	C	D
NV-004	1.66	1.42	1.629	1.349

Average: 1.5145 (Å)

B) Domain I RMSD (Å)

	Ligand-free			
	A	B	C	D
NV-004	1.16	0.983	1.218	0.875

Average: 1.059 (Å)

C) Domain II RMSD (Å) (all protein atoms)

	Ligand-free			
	A	B	C	D
NV-004	1.835	1.598	1.718	1.547

Average: 1.6745 (Å)

Supplementary Methods and Materials

Abbreviations: Rt, room temperature; Cha, 3-cyclohexyl-L-alanine; DMF, *N,N*-Dimethylformamide; DMSO, Dimethyl sulfoxide; HCTU, *O*-(6-Chlorobenzotriazol-1-yl)-*N,N,N′,N′*-tetramethyluronium hexafluorophosphate; NMM, 4-Methylmorpholine; TLC, thin layer chromatography.

A. Synthesis of NV-004 inhibitor: NV-004 (**1**) was prepared according to Scheme 1. The N^{α}-Boc protecting group of glutamine surrogate (**2**) was removed via treatment with HCl in 1,4-dioxane. Coupling of the resulting crude hydrochloride salt with Boc-Cha-OH was accomplished using HCTU and NMM in DMF to give dipeptide **3**. The same conditions were then used for removal of the N^{α}-Boc protecting group of **3** and subsequent coupling with indole-2-carboxylic acid. The *C*-terminal methyl ester was then reduced using sodium borohydride to give alcohol (**4**). Finally, oxidation of alcohol (**4**) using Dess-Martin periodinane afforded the desired aldehyde (**1**) in moderate yield.

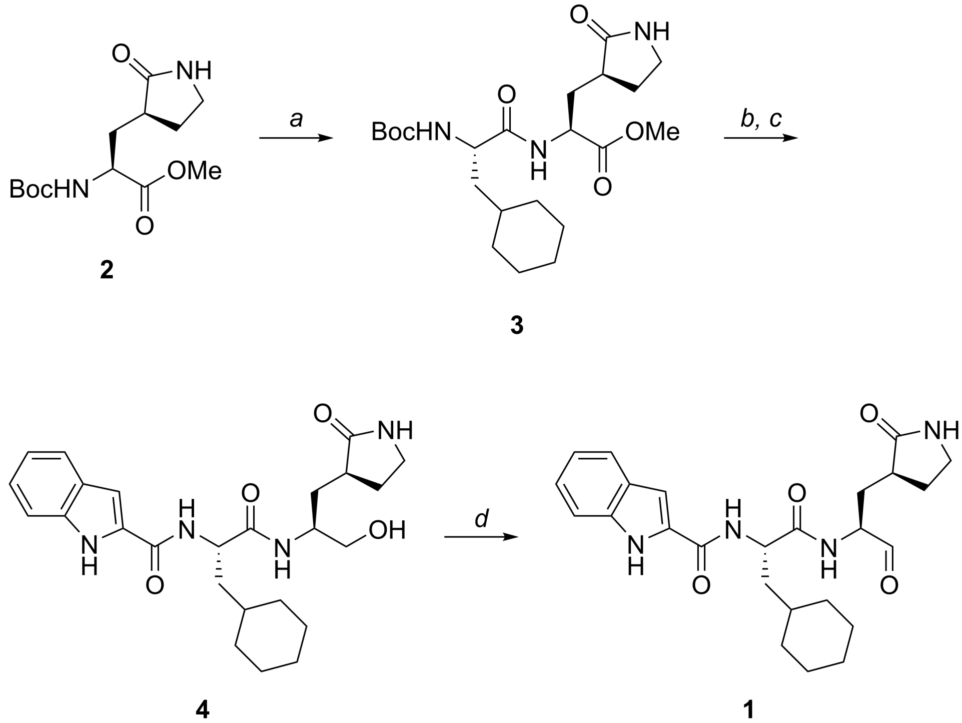

Scheme 1. Reagents and conditions. a) (i) 4 M HCl/1,4-dioxane, 0 °C to rt, 1 h; (ii) Boc-Cha-OH, HCTU, NMM, DMF, 0 °C, 1 h, 86%; b) (i) 4 M HCl/1,4-dioxane, 0 °C to rt, 1 h; (ii) indole-2-carboxylic acid, HCTU, NMM, DMF, 0 °C, 1.5 h; c) $NaBH_4$, MeOH, rt, 6.5 h, 63% over 2 steps; d) Dess-Martin periodinane, CH_2Cl_2, 0 °C to rt, 2 h, 47%.

General experimental details: Reagents were used as supplied, unless otherwise stated. Building block **2** was purchased from Angene Chemical. Solvents were purified using a LC Technical SP-1 solvent purifier system. Reactions were monitored by TLC analysis using Kieselgel 60 F_{254} aluminium-backed silica gel plates. Plates were visualized under shortwave/longwave (254/365 nm) UV light, followed by staining with vanillin, ninhydrin, or potassium permanganate stain solutions. Chromatographic purification was carried out using Kieselgel 60 (0.04 – 0.063 mm, 230-400 mesh) silica, with the eluent(s) as indicated in the text.

^{1}H and ^{13}C NMR spectra were obtained at room temperature using a Bruker AVANCE 400 spectrometer in $CDCl_3$, CD_3OD, or DMSO-d_6 as solvent. ^{1}H and ^{13}C spectra are calibrated to residual

solvent (^{1}H: δ 7.26, 3.31, or 2.50; ^{13}C: δ 77.16, 49.00, and 39.52 ppm; for $CDCl_3$, CD_3OD, and DMSO-d_6, respectively). Chemical shifts are reported in parts per million (ppm). ^{1}H NMR values are reported as chemical shift, multiplicity (where s = singlet, d = doublet, t = triplet, q = quartet, m = multiplet, and br = broad), integral, and coupling constant.
Mass spectra were obtained on a Bruker micrOTOF-QII LC-MS/MS equipped with electrospray ionisation in positive mode.

Compound 3 (JGH0015-5)

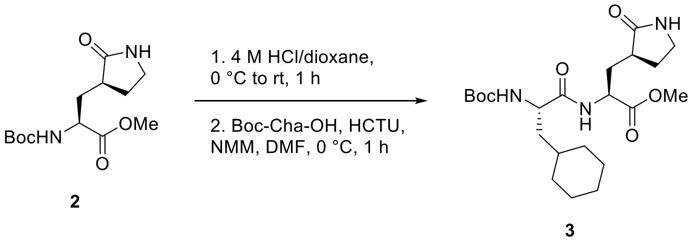

Glutamine surrogate **2** (0.20 g, 0.70 mmol) was dissolved in 4 M HCl in 1,4-dioxane (3.5 mL) at 0 °C. The reaction was warmed to rt and stirred for 1 h. The volatiles were removed under reduced pressure to give the crude HCl salt as a sticky pale-yellow oil. The crude HCl salt was used directly in the next reaction without further purification.

HCTU (0.32 g, 0.77 mmol) and NMM (0.19 mL, 1.75 mmol) were added sequentially to a stirred solution of the above crude HCl salt (0.70 mmol) and Boc-L-Cha-OH (0.19 g, 0.70 mmol) in DMF (3.5 mL) at 0 °C. The reaction was stirred at 0 °C for 1 h, quenched with 1:1 ice/sat. aq. $NaHCO_3$ (40 mL), and the mixture was extracted with EtOAc (3 x 30 mL). The combined organic extracts were washed with brine (30 mL), dried over Na_2SO_4, and concentrated under reduced pressure. The crude product was purified by flash column chromatography (silica gel, MeOH/CH_2Cl_2 1:49 to 1:19 as eluent) to give the desired product **3** (0.27 g, 86%) as a colourless solid.
^{1}H NMR ($CDCl_3$, 400 MHz): δ 7.57 (d, J = 6.7 Hz, 1H), 6.39 (s, 1H), 5.04 (d, J = 8.0 Hz, 1H), 4.54-4.49 (m, 1H), 4.28-4.22 (m, 1H), 3.71 (s, 3H), 3.36-3.27 (m, 2H), 2.41-2.35 (m, 2H), 2.23-2.16 (m, 1H), 1.92-1.79 (m, 4H), 1.72-1.61 (m, 5H), 1.42 (s, 9H), 1.39-1.33 (m, 1H), 1.28-1.10 (m, 3H), 1.00-0.85 (m, 2H).

^{13}C NMR ($CDCl_3$, 100 MHz): δ 179.8, 173.4, 172.3, 155.7, 79.9, 52.5, 51.3, 40.8, 40.5, 38.3, 34.1, 33.8, 33.4, 32.8, 28.4, 28.3, 26.6, 26.4, 26.3.

HRMS m/z (ESI+) 462.2574 $[M + Na]^+$ (calcd for $C_{22}H_{37}N_3NaO_6^+$ 462.2575).

Spectroscopic data were consistent with literature values [24]

Compound 4 (JGH0021-1)

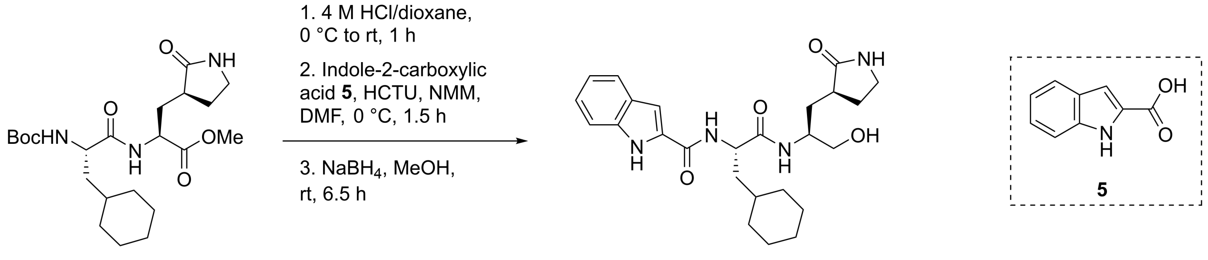

3 **4**

4 M HCl in 1,4-dioxane (1.7 mL) was added to a stirred solution of dipeptide **3** (0.15 g, 0.34 mmol) in 1,4-dioxane (1 mL) at 0 °C. The reaction was allowed to warm to rt and was stirred for 1 h. The volatiles were removed under reduced pressure to give the crude HCl salt.

HCTU (0.16 g, 0.38 mmol) and NMM (0.09 mL, 0.85 mmol) were added sequentially to a stirred solution of the above crude HCl salt and indole-2-carboxylic acid **5** (55 mg, 0.34 mmol) in DMF (1.7 mL) at 0 °C. The reaction mixture was stirred a 0 °C for 1.5 h, quenched with ice/sat. aq. $NaHCO_3$ (1:1, 30 mL) and extracted with EtOAc (2 x 30 mL). The combined organic extracts were washed with sat. aq. $NaHCO_3$ (30 mL), and brine (3 x 30 mL), dried over Na_2SO_4 and concentrated. The crude material (0.185 g) was carried forward to the next step without further purification.

Sodium borohydride (0.13 g, 3.41 mmol) was added portionwise to a stirred solution of the crude material in MeOH (14 mL) at rt. The mixture was stirred at rt for 5.5 h, additional sodium borohydride (0.13 g, 3.41 mmol) was added and the reaction was stirred for a further 1 h. Water (30 mL) was slowly added and the resulting mixture was extracted with EtOAc (3 x 30 mL). The combined organic extracts were washed with brine (20 mL), dried over Na_2SO_4, and concentrated under reduced pressure. The crude product was purified by flash column chromatography (silica gel, MeOH/CH_2Cl_2 1:19 to 1:9 as eluent) to give the desired product **4** (98 mg, 63% over 3 steps) as a colourless solid.

^{1}H NMR (CD_3OD, 400 MHz): δ 7.59 (d, J = 8.2 Hz, 1H), 7.41 (d, J = 7.40 Hz, 1H), 7.21-7.17 (m, 2H), 7.03 (dd, J = 7.4, 7.4 Hz, 1H), 4.64 (t, J = 7.6 Hz, 1H), 4.03-3.97 (m, 1H), 3.57-3.47 (m, 2H), 3.25-3.13 (m, 2H), 2.55-2.47 (m, 1H), 2.33-2.25 (m, 1H), 2.01-1.95 (m, 1H), 1.85-1.62 (m, 8H), 1.56-1.40 (m, 2H), 1.32-1.14 (m, 3H), 1.05-0.93 (m, 2H).

^{13}C NMR (CD_3OD, 100 MHz): δ 182.7, 175.4, 164.0, 138.4, 131.7, 128.9, 125.2, 122.8, 121.2, 113.0, 105.3, 65.5, 53.1, 50.6, 41.5, 40.5, 39.6, 35.6, 34.8, 33.6, 33.4, 28.9, 27.6, 27.4, 27.2.

HRMS *m/z* (ESI+) 477.2469 $[M + Na]^+$ (calcd for $C_{25}H_{34}N_4NaO_4^+$ 477.2472).

^{1}H NMR data were consistent with literature values [23].

Compound 1 (NV-004, JGH0041-1)

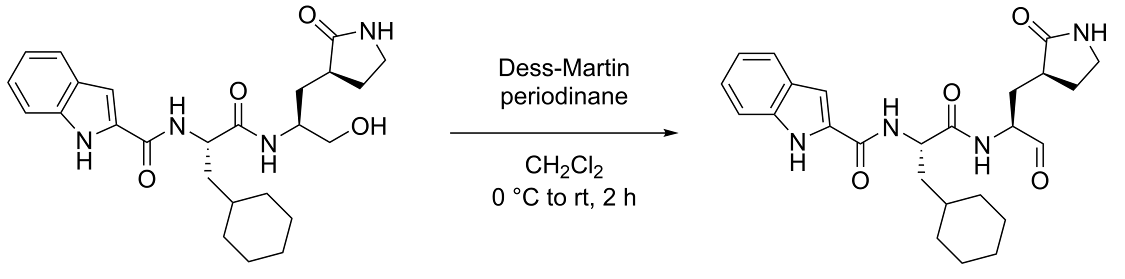

4 **1**

Dess-Martin periodinane (0.13 g, 0.30 mmol) was added to a stirred solution of alcohol **4** (0.090 g, 0.17 mmol) in CH_2Cl_2 (4 mL) at 0 °C. The reaction mixture was allowed to warm to rt and was stirred for 2 h. Sat. aq. $Na_2S_2O_3$ (20 mL) was added, the mixture allowed to stir for 5 min then extracted with EtOAc (50 mL). The organic layer was washed with sat. aq. $Na_2S_2O_3$ (20 mL), sat. aq. $NaHCO_3$ (20 mL) and brine (20 mL), dried over Na_2SO_4 and concentrated. The crude material was purified by flash column chromatography (silica gel, MeCN/EtOAc 0:1 to 2:3 as eluent) to give the desired product **5** (42 mg, 47%) as a pale yellow solid.

^{1}H NMR (DMSO-*d*$_6$, 400 MHz): *δ* 11.57 (s, 1H), 9.42 (s, 1H), 8.58 (d, *J* = 7.6 Hz, 1H), 8.48 (d, *J* = 7.9 Hz, 1H), 7.63-7.61 (m, 2H), 7.42 (d, *J* = 8.4 Hz, 1H), 7.27 (d, *J* = 1.6, 1H), 7.18 (t, *J* = 7.8 Hz, 1H), 7.03 (t, *J* = 7.5 Hz, 1H), 4.63-4.57 (m, 1H), 4.23-4.18 (m, 1H), 3.16-3.05 (m, 2H), 2.36-2.27 (m, 1H), 2.18-2.10 (m, 1H), 1.99-1.89 (m, 1H), 1.78-1.58 (m, 9H), 1.44-1.36 (m, 1H), 1.23-1.10 (m, 3H), 1.00-0.87 (m, 2H).

^{13}C NMR (DMSO-*d*$_6$, 100 MHz): *δ* 200.8, 178.3, 173.0, 161.0, 136.4, 131.3, 127.0, 123.4, 121.5, 119.7, 112.2, 103.5, 56.3, 50.7, 39.7 (obscured by solvent peak), 39.5 (obscured by solvent peak), 37.3, 33.7, 33.2, 31.8, 29.3, 27.3, 26.1, 25.8, 25.6.

HRMS *m*/*z* (ESI+) 475.2301 $[M + Na]^+$ (calcd for $C_{25}H_{32}N_4NaO_4^+$ 475.2316).

Spectroscopic data were consistent with literature values [23].

Compound 3 ¹H NMR (400 MHz, CDCl₃):

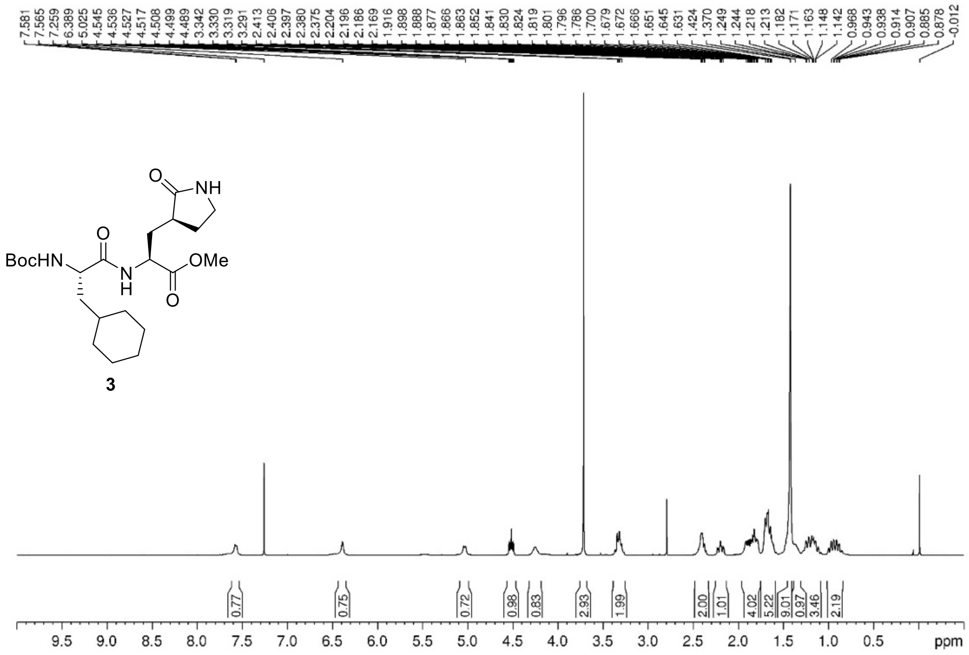

Compound 3 ¹³C NMR (100 MHz, CDCl₃):

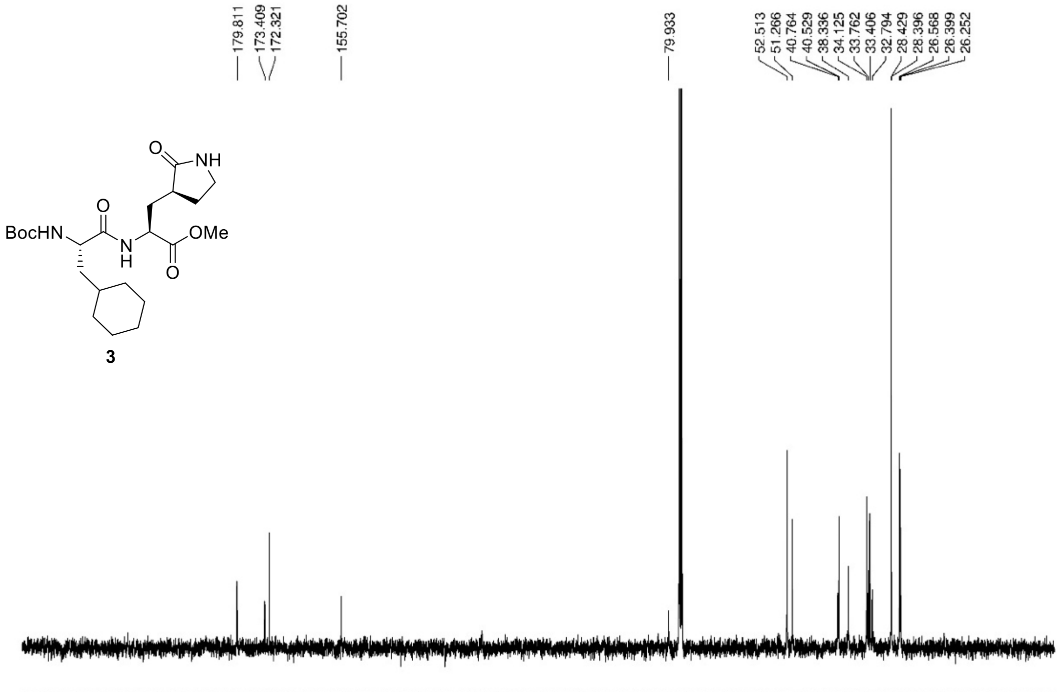

Compound 4 ^{1}H NMR (400 MHz, CD_3OD):

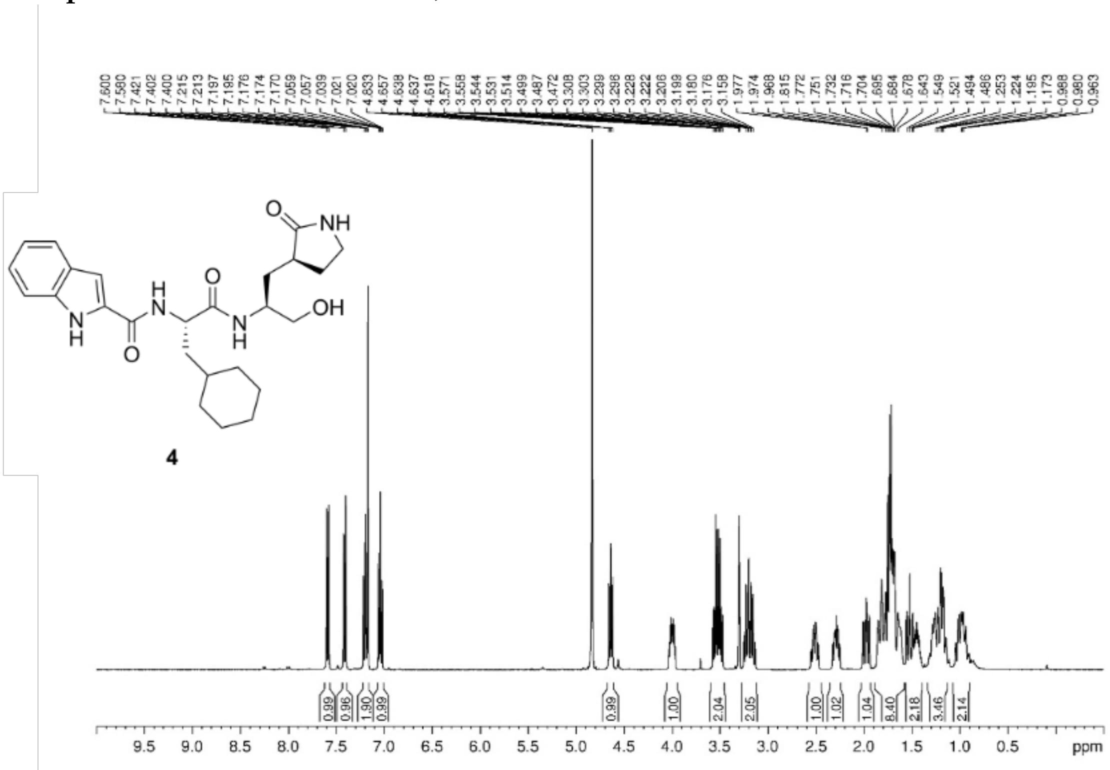

Compound 4 ^{13}C NMR (100 MHz, CD_3OD):

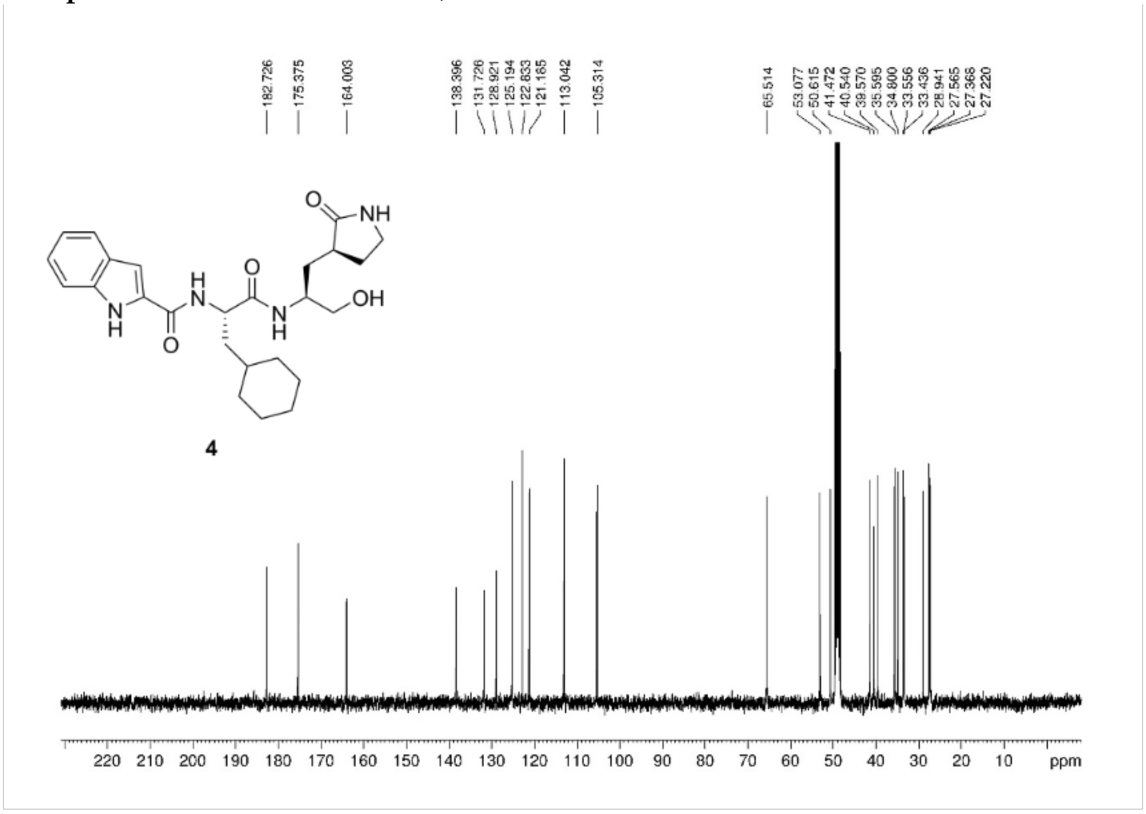

Compound 1 ^{1}H NMR (400 MHz, DMSO-d_6):

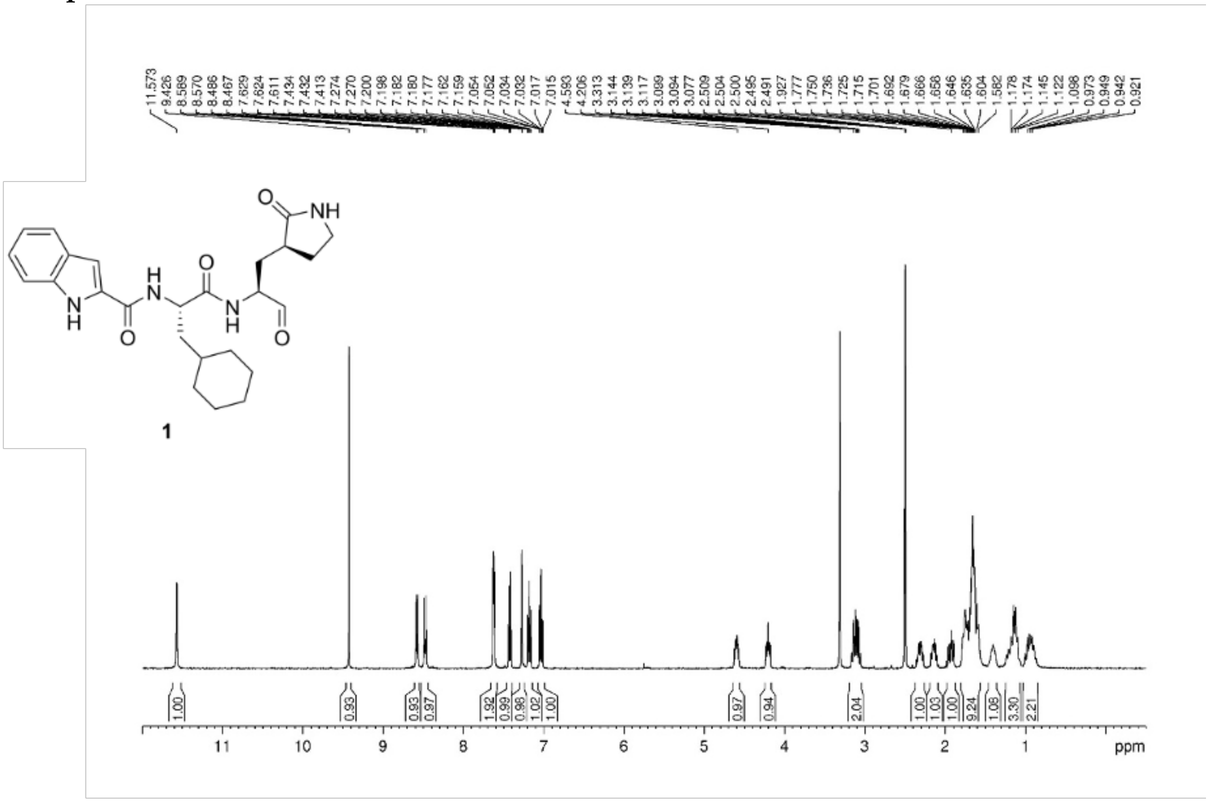

Compound 1 ^{13}C NMR (100 MHz, DMSO-d_6):

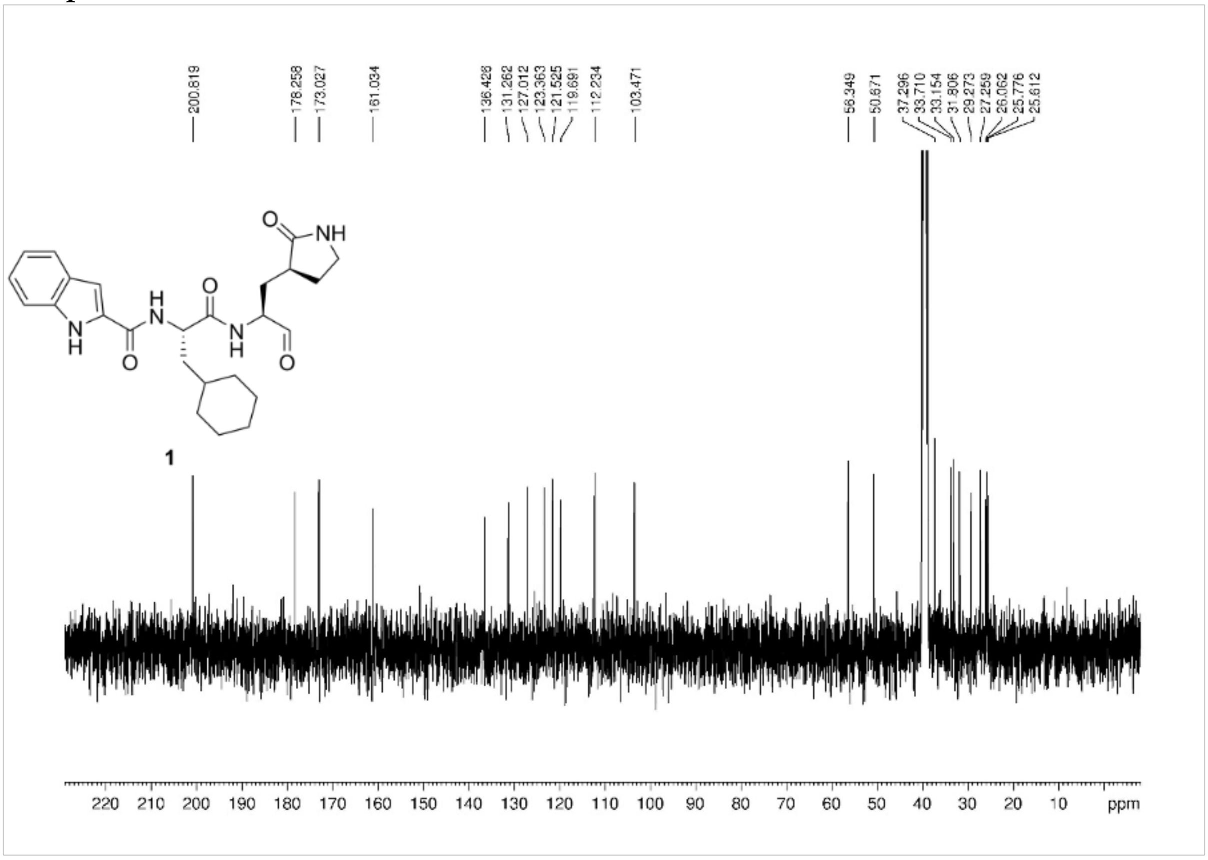

B. Synthesis of fluorescent protease substrate

The fluorescent protease substrate 5(6)-carboxyfluorescein (FAM)-LGDYELQGPEDLAK-Dabcyl was synthesized using Fmoc solid phase peptide synthesis using Fmoc-Rink amide linker attached to aminomethyl polystyrene resin.

General experimental details: RP-HPLC solvents were purchased as HPLC grade and used without further purification. Piperidine, hydroxylamine hydrochloride, imidazole, 2,4,6-trimethylpyridine, 5(6)-carboxyfluorescein, *N*,*N'*-diisopropylcarbodiimide (DIC), 1,2-ethanedithiol (EDT), triisopropylsilane (TIPS) and 4-methylmorpholine (NMM) were purchased from Sigma-Aldrich (St. Louis, Missouri). *O*-(7-Azabenzotriazol-1-yl)-*N*,*N*,*N'*,*N'*-tetramethyluronium hexafluorophosphate (HATU), Fmoc-Ala-OH, Fmoc-Arg(Pbf)-OH (Pbf = 2,2,4,6,7-pentamethyldihydrobenzofuran-5-sulfonyl), Fmoc-Asp(*t*Bu)-OH (*t*Bu = *tert*-butyl), Fmoc-Glu(*t*Bu)-OH, Fmoc-Gln(Trt)-OH (Trt = triphenylmethyl), Fmoc-Gly-OH, Fmoc-His(Trt)-OH, Fmoc-Leu-OH, Fmoc-Lys(Boc)-OH (Boc = *tert*-butoxycarbonyl), Fmoc-Phe-OH, Fmoc-Pro-OH, Fmoc-Ser(*t*Bu)-OH, Fmoc-Thr(*t*Bu)-OH, Fmoc-Trp(Boc)-OH, Fmoc-Tyr(*t*Bu)-OH, Fmoc-Val-OH and 4-[(2,4-Dimethoxyphenyl)(Fmoc-amino)methyl]phenoxyacetic acid (Fmoc-Rink amide linker) were purchased from CS Bio (Shanghai, China). 6-Chloro-1-hydroxybenzotriazole (6-Cl-HOBt) was purchased from Aapptec (Louisville, Kentucky). Aminomethyl polystyrene resin was purchased from Rapp Polymere (Tübingen, Germany). Dabcyl-OSu was purchased from Novabiochem (Darmstadt, Germany). Fmoc-Lys(Dde)-OH (Dde = 1-(4,4-dimethyl-2,6-dioxocyclohexylidene)ethyl) was purchased from AK Scientific (Union City, California). Yields refer to chromatographically homogeneous materials. Microwave reactions were carried out on a Biotage® Initiator+ Alstra™ (Uppsala, Sweden) automated peptide synthesizer. Semi-preparative/analytical RP-HPLC was performed on a Thermo Scientific (Waltham, MA) Dionex Ultimate 3000 HPLC equipped with a four channel UV detector at 210, 225, 254 and 280 nm using either an analytical column (Waters (Milford, MA) XTerra® MS C18, (5 μm; 4.6 × 150 mm) at a flow rate of 1 mL min^{-1}, or a Phenomenex® semi-preparative column (Torrance, CA), Gemini C18, (5 μm; 10 × 250 mm) at a flow rate of 4 mL min^{-1}. A suitably adjusted gradient of 5% B to 95% B was used, where solvent A was 0.1% TFA in H_2O and solvent B was 0.1% TFA in acetonitrile. LC-MS spectra were acquired using an Agilent Technologies (Santa Clara, CA) 1260 Infinity LC equipped with an Agilent Technologies 6120 Quadrupole mass spectrometer. An analytical column (Agilent ZORBAX 300SB-C3, 3.5 μm; 3.0 × 150 mm) was used at a flow rate of 0.3 mL min^{-1} using a linear gradient of 5% B to 95% B over 30 min, where solvent A was 0.1% formic acid in H_2O and solvent B was 0.1% formic acid in acetonitrile.

Synthesis of 5(6)-carboxyfluorescein (FAM)-LGDYELQGPEDLAK-Dabcyl (Scheme 2): To aminomethyl polystyrene resin (**1**, 80 mg, 0.1 mmol, loading: 1.26 mmol/g) pre-swollen in CH_2Cl_2 (5 mL, 20 min), was added 4-[(2,4-dimethoxyphenyl)(Fmoc-amino)methyl]phenoxyacetic acid (220 mg, 4 equiv., 0.4 mmol) and 6-Cl-HOBt (70 mg, 3.5 equiv., 0.35 mmol) dissolved in DMF (1.5 mL) followed by addition of DIC (62 μL, 4 equiv., 0.4 mmol). The reaction mixture was gently agitated at room temperature for 24 h. The resin was filtered and washed with DMF (3 × 3 mL) after which a negative ninhydrin test confirmed successful coupling. The *N*-Fmoc protecting group was then removed by treatment of the peptidyl resin with a solution of 20 vol% piperidine in DMF (*v*/*v*, 4 mL), and the mixture was agitated on the Biotage® Initiator Alstra for 2 × 5 min at room temperature. The resulting resin **2** was filtered and washed with DMF (3 × 3 mL).

Attachment of the *C*-terminal residue was achieved using the Biotage® Initiator Alstra by treatment of **2** with a solution of Fmoc-Dab(Dde)-OH (0.2 M in DMF, 2.5 mL, 5 equiv.), HATU (0.5 M in DMF, 0.95 mL, 4.75 equiv.), and NMM (2 M in DMF, 0.4 mL, 8 equiv.) using a single coupling cycle at 75

°C, 110 W for 5 min. The resin was filtered and washed with DMF (3 × 3 mL). The linear sequence up to [1]Leu (**Scheme 2**) was then assembled by standard Fmoc-SPPS using the previously described procedure for N^{α}-Fmoc removals and amino acid couplings. After each coupling step, unreacted amino groups were capped by treatment of the peptidyl resin with 5 M Ac_2O in DMF (0.47 mL, 2.35 equiv.) and NMM in DMF (2 M, 0.95 mL, 8 equiv.) using the Biotage® Initiator Alstra at room temperature for 10 min. The resin was filtered and washed with DMF (3 × 3 mL).
After Fmoc-deprotection on [1]Leu, the peptidyl resin (pre-swollen in CH_2Cl_2 for 20 min) was treated with 5(6)-carboxyfluorescein (112 mg, 0.3 mmol, 3 equiv.), 6-Cl-HOBt (62 mg, 0.3 mmol, 3 equiv.) and DIC (50 µL, 0.3 mmol, 3 equiv.) in DMF (1.5 mL) and agitated at room temperature for 6 h to afford intermediate **7**. The resin was filtered and washed with DMF (3 × 3 mL).
Selective Dde removal was achieved by treatment of peptidyl resin **7** (pre-swollen in CH_2Cl_2 for 30 min) with a solution of $NH_2OH{\cdot}HCl$ and imidazole (2.56 mM and 1.93 mM, respectively) in NMP/CH_2Cl_2 (6:1, 3.5 mL). The reaction mixture was gently agitated at room temperature for 5 h, followed by a second treatment with fresh reagents. The resin was filtered and washed after each round with DMF (3 × 3 mL).
To remove the 5(6)-carboxyfluorescein polymers, the peptidyl resin was treated with a solution of 20 vol% piperidine + 5 vol% formic acid in DMF (*v*/*v*, 4 mL) and the mixture was agitated for 1 × 2 min at room temperature. The resin was filtered and washed with DMF (3 × 3 mL) and repeated for an additional 5 deprotection cycles at 2 min intervals. The resin was filtered and washed with DMF (3 × 3 mL).
The Dabcyl group was installed on the side-chain amino group of [14]Lys by treatment of the peptidyl resin (pre-swollen in CH_2Cl_2 for 20 min) with Dabcyl-OSu (124 mg, 0.34 mmol, 3.4 equiv.) and 2,4,6-trimethylpyridine (93 µL, 0.7 mmol, 7 equiv.) in DMF (1.5 mL). The reaction mixture was agitated at room temperature for 30 min, and the resin was filtered and washed with DMF (3 × 3 mL).
Global deprotection and resin cleavage was achieved by treatment of the peptidyl resin with a mixture of TFA/H_2O/TIPS/EDT (94:2.5:2.5:1, *v*/*v*/*v*/*v*, 10 mL) for 2 h. The filtrate was partially concentrated under a gentle stream of N_2, then cold diethyl ether was then added to form a precipitate. The mixture was centrifuged, and the solution was carefully decanted off and discarded, before dissolving the solid pellet in H_2O:acetonitrile (1:1, *v*/*v*, 25 mL) containing 0.1% TFA, and lyophilised to afford crude peptide **8**.

Crude **8** was purified batchwise by semi-preparative RP-HPLC on a Phenomenex® Gemini C18 column (10 × 250 mm, 5 µm) using a linear gradient of 5% to 95% over 95 min (*ca.* 1% B/min) with a flow rate of 4 mL/min. Fractions were collected at 0.2 min intervals and analysed by ESI-MS and RP-HPLC. Fractions identified with correct *m*/*z* were combined and lyophilised to afford the pure *title compound* **8** as a red amorphous solid (60 mg, 27% yield based on 0.1 mmol scale), t_R = 42.5 min and 43.1 min, >99% purity by HPLC.

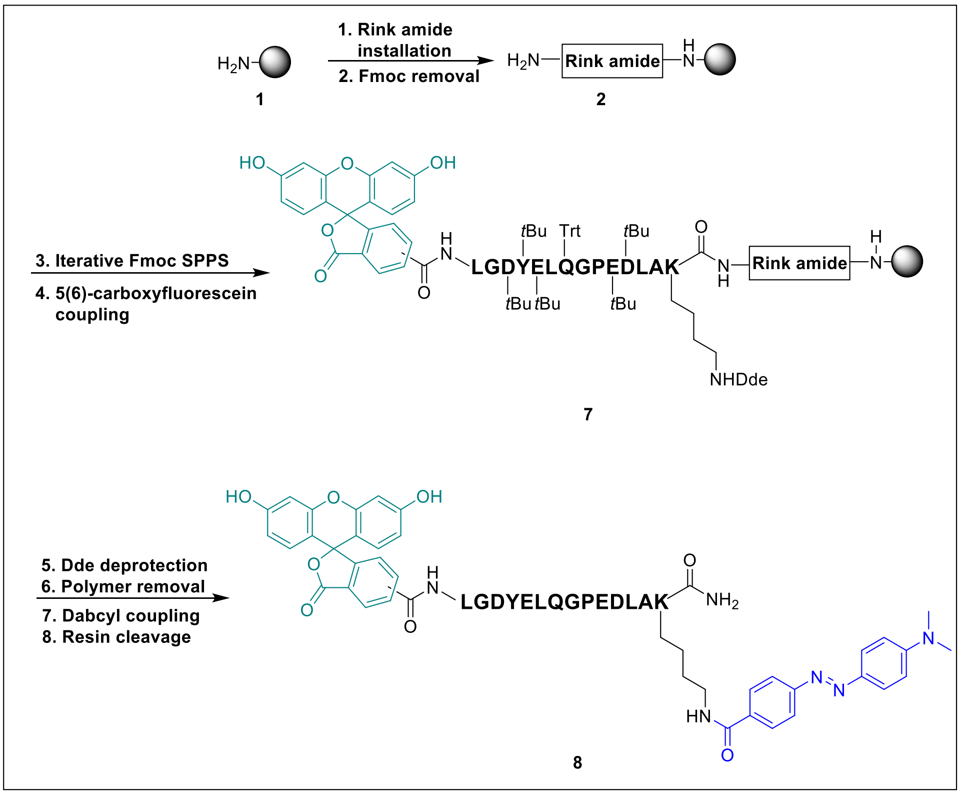

Scheme 2. Synthesis of 5(6)-carboxyfluorescein (FAM)-LGDYELQGPEDLAK-Dabcyl

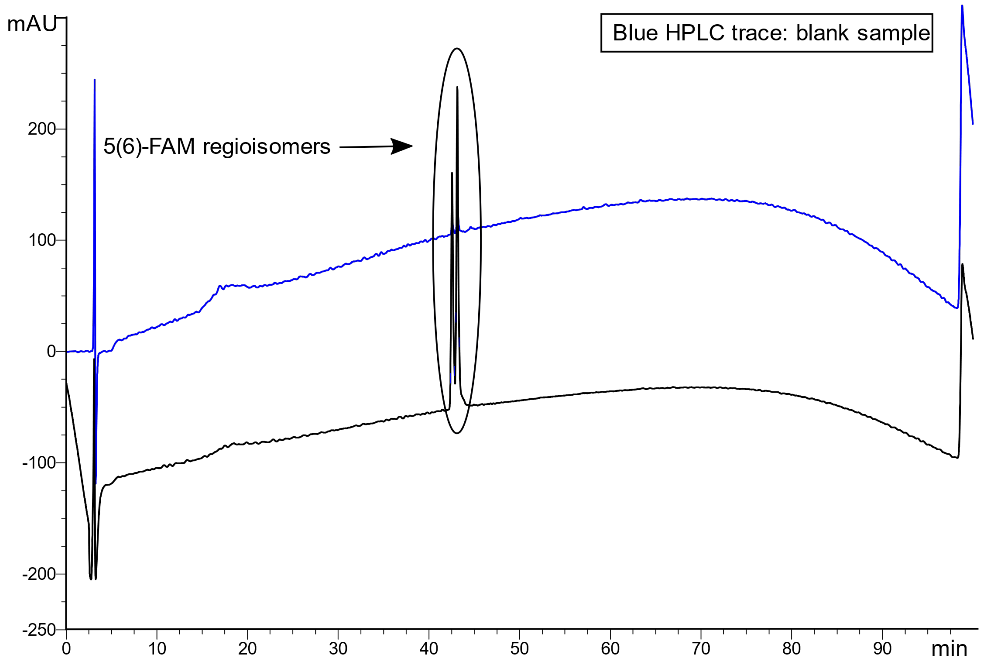

Analytical RP-HPLC chromatogram for purified peptide 8; t_R = 42.5 and 43.1 min (>99% purity as judged by peak area of RP-HPLC at 210 nm). Chromatographic separations were performed on a Thermo Scientific (Waltham, MA) Dionex Ultimate 3000 HPLC using a Phenomenex® Gemini-NX C18 column (5 µm; 4.6 × 250 mm) and a linear gradient of 5-95% B over 90 min at room temperature, *ca*. 1% B per min at a flow rate of 1.0 mL/min. Buffer A: H_2O containing 0.1% TFA (*v*/*v*); Buffer B: acetonitrile containing 0.1% TFA (*v*/*v*).

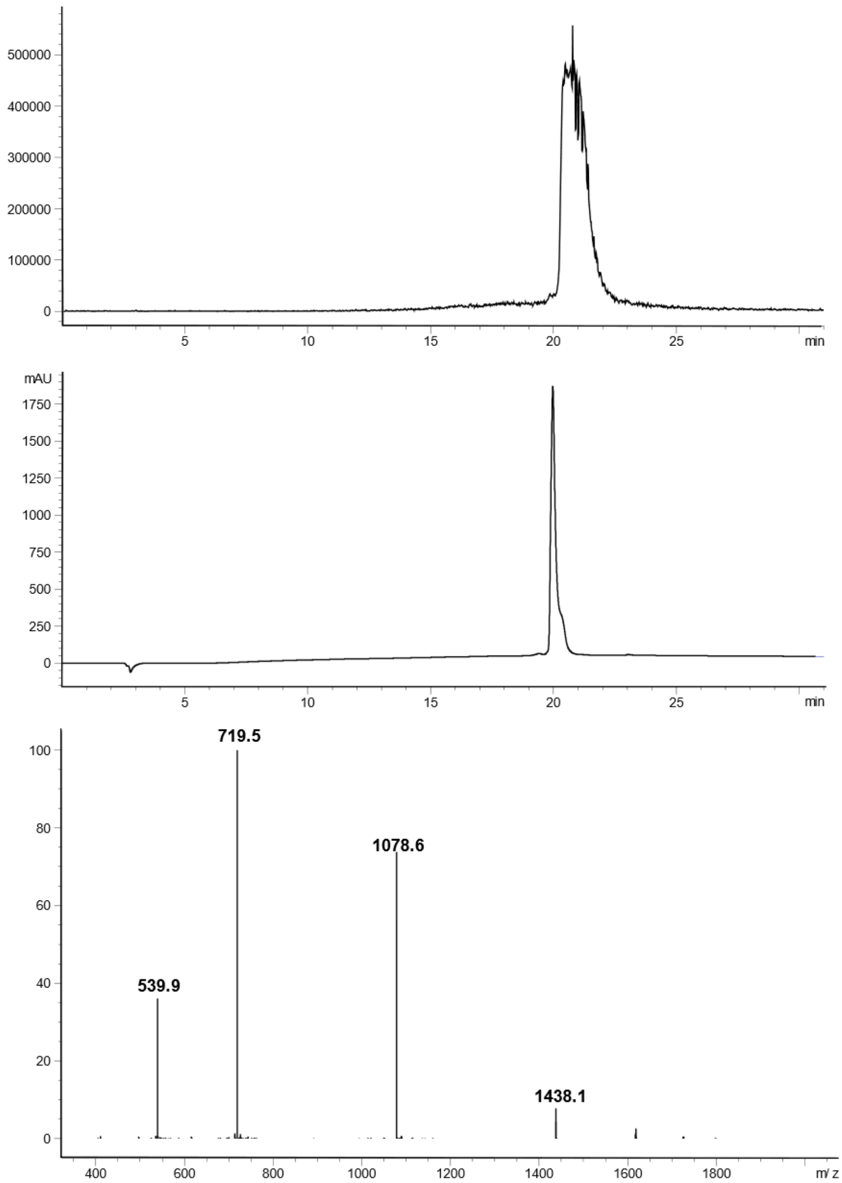

LC-MS of purified peptide 8; ion polarity positive operating at a nominal accelerating voltage of 70 eV. (*m/z* $[M+2H]^{2+}$ calcd: 1079.14; found: 1078.6; $[M+3H]^{3+}$ calcd: 719.7; found 719.5; $[M+4H]^{4+}$ calcd: 540.1; found: 539.9; $[2M+3H]^{3+}$ calcd: 1438.5; found: 1438.1. Mass deconvolution calculated at 2155.43 Da with standard deviation of 0.21; theoretical mass calculated at 2156.29 Da. LC-MS was performed on an Agilent Technologies 6120 Quadrupole mass spectrometer using an Agilent C3 column, (3.5 μm; 3.0 × 150 mm) and a linear gradient of 5-95% B over 30 min at room temperature, *ca*. 3% B per min at a flow rate of 0.3 mL/min. Buffer A: H_2O containing 0.1% formic acid (*v/v*); Buffer B: acetonitrile containing 0.1% formic acid (*v/v*).
